# Most large structural variants in cancer genomes can be detected without long reads

**DOI:** 10.1038/s41588-023-01540-6

**Published:** 2023-11-09

**Authors:** Zi-Ning Choo, Julie M. Behr, Aditya Deshpande, Kevin Hadi, Xiaotong Yao, Huasong Tian, Kaori Takai, George Zakusilo, Joel Rosiene, Arnaud Da Cruz Paula, Britta Weigelt, Jeremy Setton, Nadeem Riaz, Simon N. Powell, Klaus Busam, Alexander N. Shoushtari, Charlotte Ariyan, Jorge Reis-Filho, Titia de Lange, Marcin Imieliński

**Affiliations:** 1 New York Genome Center, New York, NY USA; 2 Department of Pathology and Laboratory Medicine, Weill Cornell Medicine, New York, NY USA; 3 Tri-institutional MD PhD Program, Weill Cornell Medicine, New York, NY USA; 4 Physiology and Biophysics PhD Program, Weill Cornell Medicine, New York, NY USA; 5 Tri-institutional PhD Program in Computational Biology and Medicine, New York, NY USA; 6 Perlmutter Cancer Center, NYU Grossman School of Medicine, New York, NY USA; 7 Laboratory of Cell Biology and Genetics, Rockefeller University, New York, NY USA; 8 Memorial Sloan Kettering Cancer Center, New York, NY USA; 9 Department of Pathology, NYU Grossman School of Medicine, New York, NY USA

**Keywords:** Genomics, Computational biology and bioinformatics, Genome assembly algorithms

## Abstract

Short-read sequencing is the workhorse of cancer genomics yet is thought to miss many structural variants (SVs), particularly large chromosomal alterations. To characterize missing SVs in short-read whole genomes, we analyzed ‘loose ends’—local violations of mass balance between adjacent DNA segments. In the landscape of loose ends across 1,330 high-purity cancer whole genomes, most large (>10-kb) clonal SVs were fully resolved by short reads in the 87% of the human genome where copy number could be reliably measured. Some loose ends represent neotelomeres, which we propose as a hallmark of the alternative lengthening of telomeres phenotype. These pan-cancer findings were confirmed by long-molecule profiles of 38 breast cancer and melanoma cases. Our results indicate that aberrant homologous recombination is unlikely to drive the majority of large cancer SVs. Furthermore, analysis of mass balance in short-read whole genome data provides a surprisingly complete picture of cancer chromosomal structure.

## Abstract (web summary)

JaBbA v1 pinpoints the ‘loose ends’ of large (>10-kb) unmapped structural variants in short-read DNA sequencing, suggesting that about 90% of cancer chromosomal alterations outside centromeres are resolvable with short reads and that long reads will primarily improve calling of smaller somatic variants.

## Main

It is widely thought that short-read sequencing (SRS), which usually generates ≤150-bp reads, has limited sensitivity for mapping cancer structural variants (SVs; copy number (CN) alterations and rearrangements) owing to the many homologous sequences in the human genome^1^. Indeed, more than two-thirds of the human genome consists of repetitive sequences^2^, including transposable elements, satellites and telomeres. SVs that rearrange long homologous repeats are likely to be missed by SRS.

Cancer whole-genome profiling efforts have been carried out almost exclusively with SRS^3–5^. Hence, little is known about the nature and burden of cancer SVs missed by SRS. While most cancer rearrangements detected with SRS have negligible breakend homology^3,6–8^, it is also unknown whether additional homologous recombination-driven mutational processes govern the evolution of rearrangements that are undetectable by SRS^1,9^.

Owing to mass balance, every copy of every segment in a genome must either have both a left and right neighbor or reside at a chromosome end. Because rearrangements appose previously distant segment ends to create new junctions, CN alterations and rearrangements are physically coupled in the cancer genome; most CN alterations involve a rearrangement, and many rearrangements are associated with a CN alteration^4,10–13^.

**Fig. 1:**
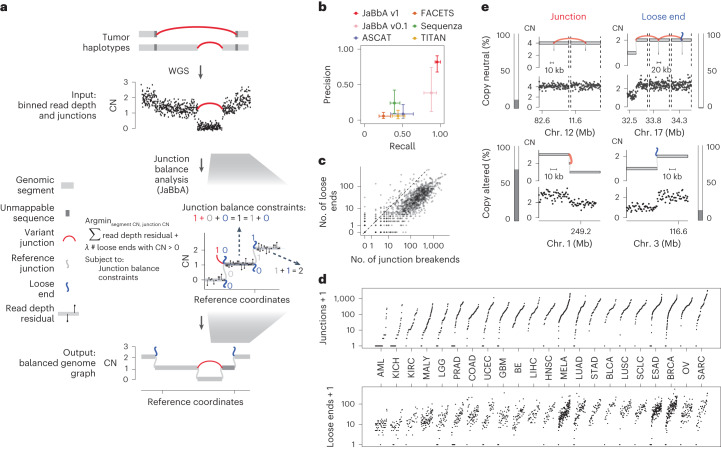
Mass balance violations in cancer genomes. **a**, Schematic for integrated SV detection in JaBbA. Rearranged tumor haplotypes (top) comprise genomic segments connected by variant DNA junctions. These haplotypes produce read depth changes (scatterplot) and variant adjacencies (red edges) in SRS whole-genome profiles (second track from top). JaBbA solves a mixed-integer program to identify the balanced genome graph that optimally explains the input (read depth and adjacencies; third track, right). Graph edges comprise reference or variant junctions and loose ends. Loose ends are placeholder edges that represent local violations of mass balance, which can occur at the breakends of junctions that are missing from the data (Methods). **b**, Precision and recall of SV breakend detection by JaBbA v1 in comparison to other state-of-the-art CN inference algorithms (JaBbA v0.1, ASCAT v2.5.2 (ref. ^14^), FACETS v0.6.2 (ref. ^17^), Sequenza v3.0 (ref. ^16^) and TITAN v1.28 (ref. ^15^)) in a simulated dataset of 500 samples. Points show medians across all samples, and error bars show the IQR. **c**, Somatic loose end count (*y* axis) versus somatic junction breakend count (*x* axis) identified by JaBbA across a pan-cancer cohort of 1,330 high-purity matched tumor–normal tissue samples. The line shows *x* = *y*, and points correspond to breakend counts + 1 on a log_10_ scale. **d**, Number of junction breakends (top) and number of loose ends (bottom) by tumor type. Counts are plotted on a log scale after adding 1. AML, acute myeloid leukemia; KICH, kidney chromophobe; KIRC, kidney renal clear cell carcinoma; MALY, malignant lymphoma; LGG, low-grade glioma; PRAD, prostate adenocarcinoma; COAD, colon adenocarcinoma; UCEC, uterine corpus endometrial carcinoma; GBM, glioblastoma multiforme; BE, Barrett’s esophagus; LIHC, liver hepatocellular carcinoma; HNSC, head and neck squamous cell carcinoma; MELA, melanoma; LUAD, lung adenocarcinoma; STAD, stomach adenocarcinoma; BLCA, bladder carcinoma; LUSC, lung squamous cell carcinoma; SCLC, small cell lung cancer; ESAD, esophageal adenocarcinoma; BRCA, breast carcinoma; OV, ovarian adenocarcinoma; SARC, sarcoma. **e**, Fractions and examples of copy-neutral and copy-altered breakends associated with junctions and/or loose ends. Outer bar plots show the fractional contribution of each of the four breakend classes (e.g. copy-altered loose ends in the bottom right) to the total number of SV breakends detected by JaBbA. In each subpanel, the top track shows the balanced genome graph with plot elements as in **a** and the bottom track shows binned purity- and ploidy-transformed tumor read depth. Source data

This coupling can be formalized as ‘junction balance constraints’ on a graph of genomic segments and their junctions^4^ (Fig. 1a). These constraints state that the CN of each genomic segment is equal to the CN of the junctions connecting to its left and right sides. Enforcing these and other constraints within a statistical model enables the inference of balanced genome graphs and high-fidelity CN profiles from whole-genome SRS data, as shown with our previously published JaBbA (v0.1) algorithm^4^.

JaBbA’s statistical model allows for ‘loose ends’, which are ‘placeholder’ adjacencies that allow the graph to satisfy junction balance while violating mass balance (Fig. 1a). Loose ends allow JaBbA to be robust to missing data but also represent hypotheses about unmapped junctions. We reasoned that analysis of loose ends in JaBbA could be used to test the completeness of cancer genome reconstructions from SRS and assess the nature of missing SVs in SRS profiles. In particular, we focused on large (>10-kb) SVs that give rise to clonal chromosomal alterations in cancers (referred to as SVs below for brevity, unless otherwise qualified). Our goal was to understand the impact of mutational processes that specifically rearrange repetitive sequences, including aberrant homologous recombination, on cancer chromosomal structure.

## Results

### JaBbA v1 outperforms previous CN algorithms

**Extended Data Fig. 1:**
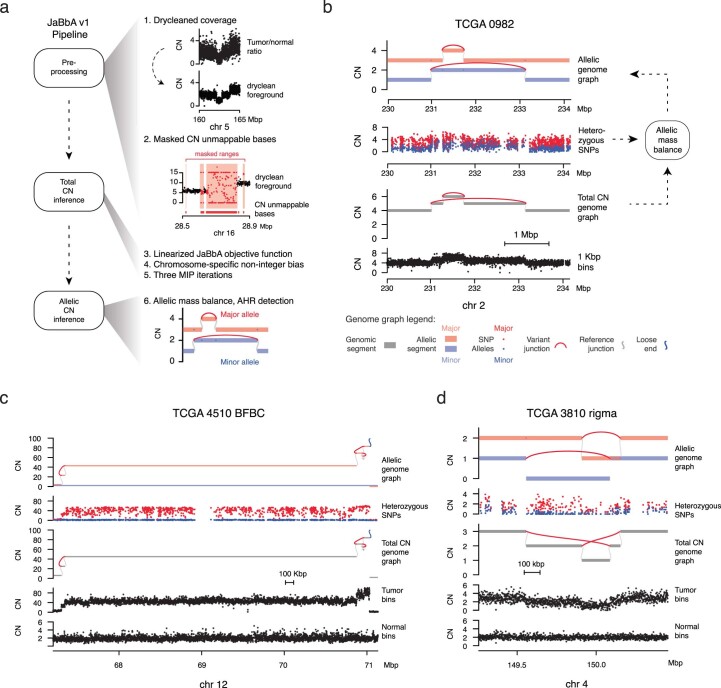
Improvements to JaBbA. (a) Overview of JaBbA pipeline and improvements in JaBbA v1 (see Methods for details). AHR, allelic homologous recombination. (b) Example of allelic CN inference in TCGA case 0982. After fitting total CN, mass balance constraints are then applied to identify the optimal CN assignment to the nodes and edges of an allelic genome graph, that is, one that maximally explains purity-ploidy transformed allelic counts at heterozygous SNPs. The vertices and edges of the resulting balanced allelic graph are annotated with allele-specific CNs. (c) Example of a balanced allelic graph corresponding to a breakage-fusion-bridge cycle variant in a TCGA sample. Genome graph legend as for panel b. (d) Example of balanced allelic graph corresponding to a rigma variant in TCGA sample 3810. In b-d allelic and total read depth is purity / ploidy transformed (see Methods). Genome graph legend as for panel b. Source data

**Extended Data Fig. 2:**
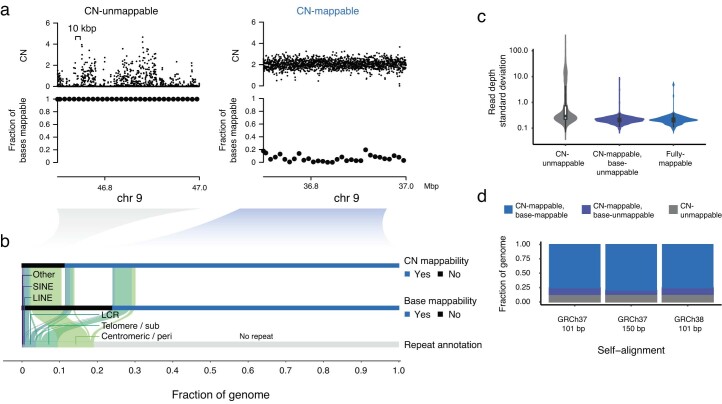
Defining the CN-mappable genome. (a) Example of read depth in the matched normal sample of TCGA case 6864 at a locus with a high density of unmappable bases (left, CN-unmappable) and at a locus with low density of unmappable bases (right, CN-mappable). In both panels, the top track shows purity / ploidy transformed read depth per 1 kbp bin (scaled to CN units) and the bottom track shows the density of unmappable bases per 10 kbp bin. The expected purty / ploidy transformed read depth for all bins is two as this is a non-neoplastic sample. (b) Fraction of the genome classified as CN-unmappable (top), base-unmappable (middle), and fully-mappable (bottom). CN-unmappable regions refer to genomic positions surrounded by ≥90% multi-mapping bases (bases with multiple alignments at a length of 101 bp and > 96% homology) in their 1 kbp vicinity. Base-unmappable regions refer to bases pairs falling within CN-unmappable regions, in addition to base pairs in lower density regions that are multi-mapping. The remainder of the genome is comprised of fully-mappable bases. SINE, short interspersed nuclear element. LINE, long interspersed nuclear element. LCR, low copy repeat, (c) Distribution of the standard deviation of read depth within 10 kbp bins for normal samples in CN-unmappable, base-unmappable, and fully-mappable regions in normal samples (n = 1000 CN-unmappable, 1000 base-unmappable, 1000 fully-mappable tiles sampled across 100 normal short-read whole genome sequencing profiles). Box plot: line (median), body (IQR), whiskers (1.5 times IQR). (d) The fraction of the genome designated CN-unmappable, base-unmappable, and fully-mappable using either 101 bp or 150 bp self-alignment, and either GrCh37 or GrCh38 as the reference genome. The fraction of the genome that is CN-unmappable is 13% in all three cases shown.

We enhanced our previous JaBbA (v0.1; ref. ^4^) model with several methodological innovations to increase robustness to read depth waviness, improve algorithm convergence and enforce junction balance for allele-specific as well as total CN (Extended Data Fig. 1a–d and Methods). We also rigorously defined ‘CN-unmappable’ regions in the genome as positions surrounded by >90% repetitive bases in their 1-kb vicinity. CN-unmappable regions accounted for 13% of the genome (across read lengths and genome builds), primarily comprised regions in or around telomeres and centromeres, and showed high variance in read depth across a panel of diploid normal samples (Methods and Extended Data Fig. 2). We then limited analysis with the updated model (JaBbA v1) to the 87% of the human genome that was CN-mappable.

**Extended Data Fig. 3:**
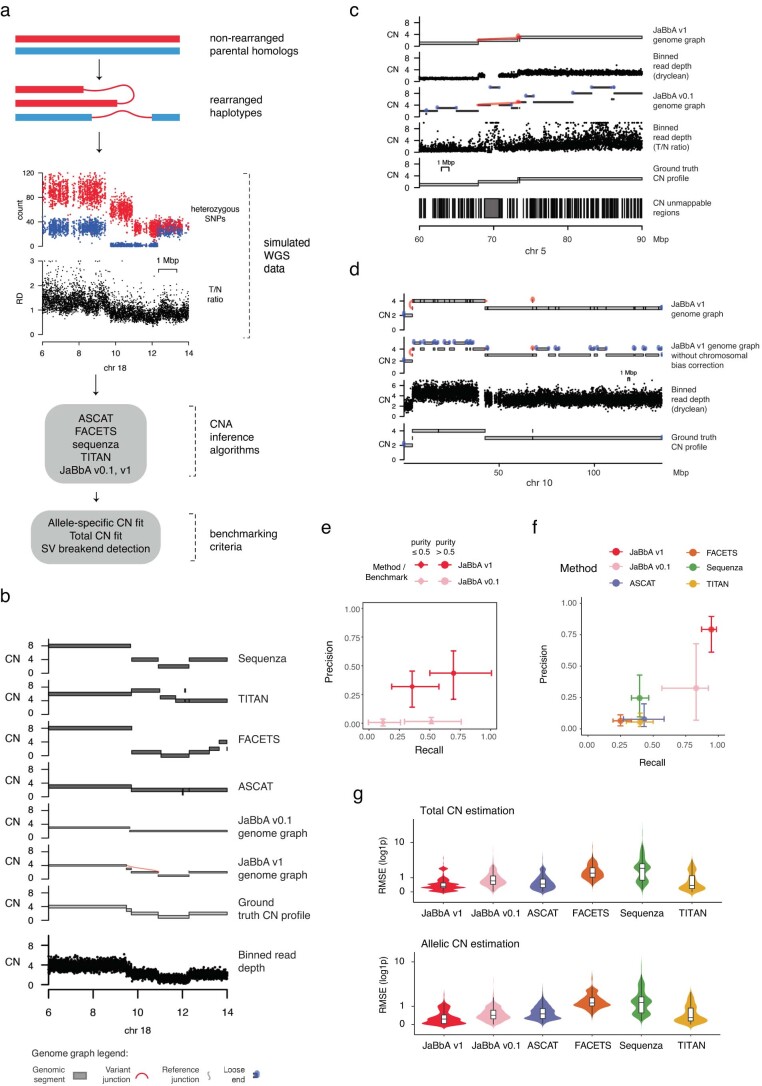
Benchmarking CN estimation and breakend detection in cancer genomes. (a) Benchmarking pipeline for simulating SRS data and testing the accuracy of inferred CN profiles. Junctions are simulated to rearrange parental haplotypes of the phased NA12878 Platinum genome yielding a rearranged and copy-altered cancer genome (top). Simulated haplotypes are sampled to generate 1 Kbp bins of total read depth, allelic counts at heterozygous SNPs, and set of junctions according to a sampled purity and ploidy value (second from top). These data are then analyzed by the CN algorithms shown in the bottom panel and results are compared to the ground truth allelic and total CN profile (bottom). See Methods for additional details. (b) Example of a simulated locus, along with inferred total CN by each algorithm (top panels), the simulated ground truth CN profile (second from bottom), and tumor purity / ploidy transformed read depth (bottom, see Methods). (c) Example of genome graphs inferred by JaBbA v1 and JaBbA v0.1 for another locus in one of the 500 simulated samples. Tracks from top to bottom show the genome graph inferred by JaBbA v1, the dryclean foreground, the graph inferred by JaBbA v0.1, the tumor/normal read depth ratio, the ground truth copy CN, and the location of CN-unmappable ranges masked in JaBbA v1. Both dryclean foreground and tumor/normal ratio are purity / ploidy transformed (see Methods). Genome graph legend same as for panel b. (d) Comparison of graphs inferred by JaBbA v1 and JaBbA v0.1, for another simulated tumor sample and locus. Tracks from top to bottom show graph inferred by JaBbA v1, graph inferred by JaBbA v0.1, read depth, and ground truth CN. Both dryclean foreground and tumor/normal ratio are purity / ploidy transformed, that is in CN units (see Methods). Genome graph legend same as for panel b. (e) Precision and recall for the detection as loose ends of SVs missing from the junction input to JaBbA (n = 500 simulated tumors). JaBbA v1 and v0.1 are compared, stratified by inferred sample purity. Points show median and error bars show IQR.(f) Overall precision and recall of SV breakend calls by JaBbA and other CN inference algorithms for all simulated samples, including those with low ( < 0.5) purity (n = 500 simulated tumors). Points show median and error bars show IQR. (g) Root mean square error for total (top) and allele-specific (bottom) CN by JaBbA and other CN inference algorithms (n = 500 simulated tumors). Box plots: line (median), body (IQR), whiskers (1.5 IQR). Source data

To assess the accuracy of JaBbA v1 for SV breakend detection in CN-mappable regions, we simulated 500 SRS whole-genome profiles comprising binned (1-kb) read depth, single nucleotide polymorphism (SNP) read counts and SV junctions (Extended Data Fig. 3a–d and Methods). In these simulations, JaBbA v1 loose ends showed substantially higher precision (median of 43% versus 5%) and recall (median of 70% versus 54%) than JaBbA v0.1 loose ends for missing CN-mappable SVs in high-purity (>0.5) cancer genomes (Extended Data Fig. 3e). JaBbA v1 also showed markedly improved accuracy for overall CN-mappable SV breakend inference relative to both JaBbA v0.1 and four state-of-the-art cancer CN inference algorithms (ASCAT^14^, TITAN^15^, Sequenza^16^ and FACETS^17^) (Extended Data Fig. 3f), particularly for high-purity samples (median precision of 82% (68–91%) and median recall of 96% (93–100%), with the interquartile range (IQR) in parentheses) (Fig. 1b). JaBbA v1 also accurately estimated both total and allelic CN (Extended Data Fig. 3g), suggesting that JaBbA v1 is a state-of-the-art algorithm for the inference of CN and missing SVs in cancer genomes.

### Pan-cancer landscape of loose ends

We next applied JaBbA v1 to 1,330 high-purity tumor and matched normal SRS profiles previously analyzed in Hadi et al.^4^ (see Methods for details), identifying 154,322 (clonal and somatic) junctions (median of 63 per tumor sample) and 48,835 somatic loose ends (median of 21 per tumor sample). The somatic loose end burden per sample varied across a 200-fold range and was correlated (Spearman *R*^2^ = 0.68) with the junction burden (Fig. 1c,d).

**Extended Data Fig. 4:**
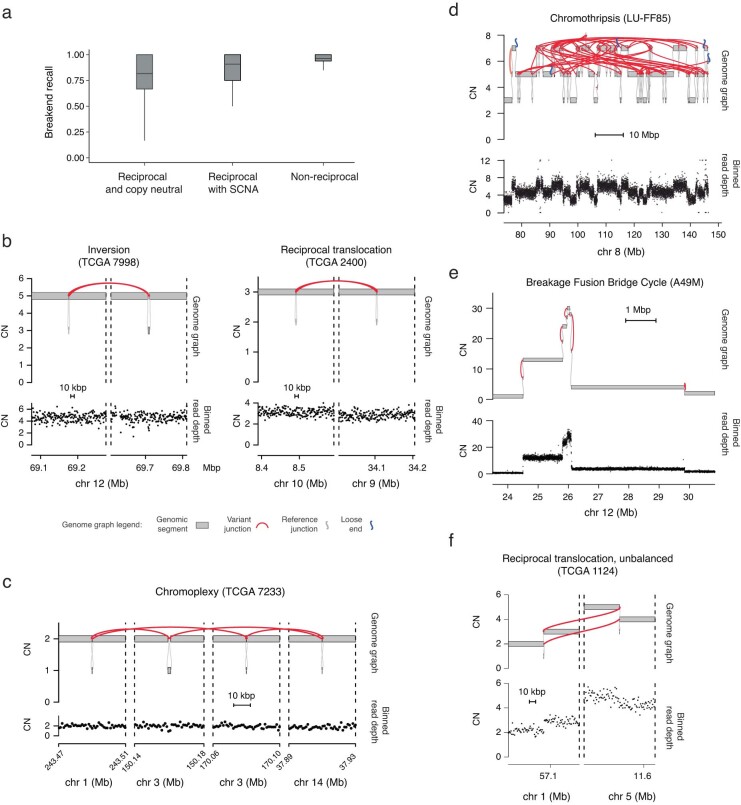
Examples of copy-neutral and copy-altered SVs detected by JaBbA. (a) Fraction of breakends recovered by JaBbA in simulated genomes, by breakend class (described in Extended Data Fig. 3). Box plot: line (median), body (IQR), whiskers (1.5 times IQR) (b-c) Examples of simple (inversion, translocation) and complex (chromoplexy) copy-neutral reciprocal SVs. (d,e) Examples of complex non-reciprocal SVs associated with CN alterations. (f) Example of a copy-altered reciprocal translocation. In each example in panels b-f, the top graph shows the JaBbA genome graph and the bottom graph shows the binned purity / ploidy transformed tumor read depth. Genome graph legend for plots b-f is shown in panel b. Source data

Junction breakends may be reciprocal, meaning that they are near (within 10 kb) of another breakend with opposite orientation. Reciprocal breakends are usually copy-neutral (Fig. 1e, top left) which makes them difficult to detect through classic CN analyses. JaBbA’s bookkeeping of mass balance across segments and junctions enables sensitive detection of reciprocal and nonreciprocal SVs at both copy-neutral and copy-altered genomic regions (Extended Data Fig. 4a–e). Across cancer, we found that most (85%) cancer junctions were both nonreciprocal and copy-altered (Fig. 1e, bottom left). Such junctions can arise from inherently nonreciprocal SVs, such as simple deletions, or begin as reciprocal translocations that undergo subsequent loss or gain of one of the derivative alleles (Extended Data Fig. 4f). Like somatic junction breakends, somatic loose ends were predominantly (92%) copy-altered (Fig. 1e, bottom right), although copy-neutral loose ends were also identified (Fig. 1e, top right). Taken together, these results suggest that loose ends arise by breakage and repair mutational processes similar to those generating junction breakends.

### Loose ends harbor repetitive and foreign sequences

**Fig. 2:**
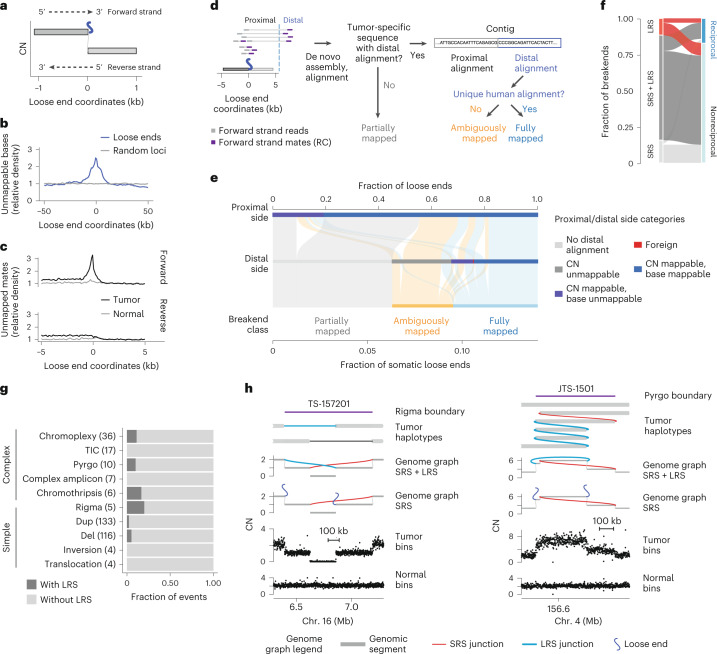
Loose ends pinpoint missing cancer SVs. **a**, Loose end coordinates are centered at each loose end and increase in the 5′ to 3′ direction along the forward strand. For a loose end arising from the right side of its associated reference genomic segment (that is, the side with larger reference genomic coordinates), the forward strand is the positive reference strand, that is, the strand with increasing reference coordinates along its 5’ to 3’ direction. Conversely, for a loose end arising from the left side of its associated reference genomic segment, the forward strand is the negative reference strand. **b**, Density of unmappable bases around loose ends (Methods). **c**, Density of uniquely mapping reads with unmapped (i.e. non-uniquely aligning) mates around loose ends. **d**, Subclassification of loose ends based on local assembly and consensus alignment (Methods). RC, reverse complement. **e**, Alluvial plot showing each loose end class (bottom row) and the mappability tier of the distal (middle row) and proximal (top row) ends of breakend sequences obtained through local assembly or consensus alignment. **f**, Alluvial plot comparing SRS and LRS breakend calls. The fraction of breakends identified by LRS only, SRS only and both platforms (LRS and SRS) is shown (left), stratified by whether the breakend was reciprocal to another breakend in the same sample (right). LRS breakends were taken from tumor-specific junctions found by at least two of four LRS SV callers (SVIM^49^, cuteSV^50^, Sniffles2 (ref. ^51^) and SAVANA^52^). SRS breakends comprise junction breakends and loose ends in the JaBbA v1 genome graph. **g**, Stacked barplots showing the fraction of complex SVs called from genome graphs with versus without the addition of LRS junctions. DEL, deletion; DUP, duplication; TIC, templated insertion chain. **h**, Examples of a rigma (left) and pyrgo (right) identified by LRS and missed by SRS. Tracks from top to bottom show tumor haplotype reconstructions, a genome graph with SRS and LRS junctions, a genome graph with only SRS junctions, and purity- and ploidy-transformed read depth. Pyrgo and rigma boundaries are delineated by a purple line at the top of each plot. Source data

**Extended Data Fig. 5:**
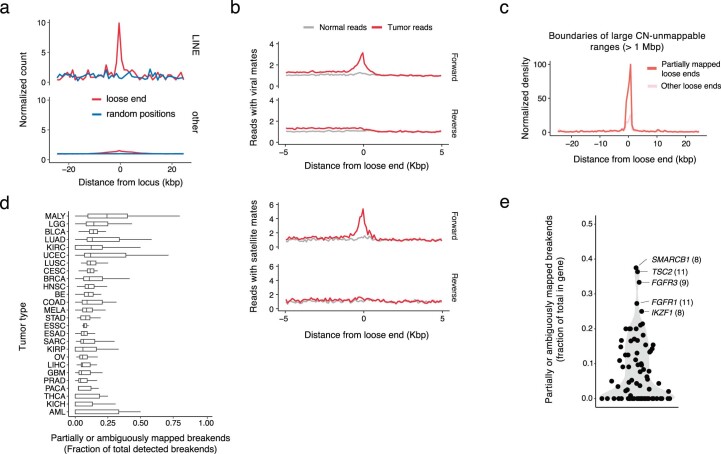
Genomic context of loose ends. (a) Density of unmappable bases overlapping RepeatMasker-annotated LINE elements (top) and repeat classes in standard loose end coordinates (b) Density of reads mated to viral sequences (top) and satellite repeats (bottom) in the vicinity of loose ends. See Fig. 2a for definition of forward and reverse strands. (c) Enrichment of large ( > 1 Mbp) CN-unmappable segment boundaries near partially mapped breakends (red) in comparison to other loose ends (pink). (d) Fraction of breakends in CN-mappable regions of the genome not fully resolved by SRS per sample, grouped by tumor type. Box plot: line (median), body (IQR), whiskers (1.5 times IQR) (n = 48 AML, Acute Myeloid Leukemia; 20 BLCA, Bladder Urothelial Carcinoma; 127 BRCA, Breast Invasive Carcinoma; 15 CESC, Cervical Squamous Cell Carcinoma and Endocervical Adenocarcinoma; 57 COAD, Colon Adenocarcinoma; 186 ESAD, Esophageal Adenocarcinoma; 43 GBM, Glioblastoma Multiforme; 30 HNSC, Head and Neck Squamous Cell Carcinoma; 40 KICH, Kidney Chromophobe Carcinoma; 24 KIRC, Kidney Renal Clear Cell Carcinoma; 19 KIRP, Kidney Renal Papillary Cell Carcinoma; 41 LGG, Brain Lower Grade Glioma; 28 LIHC, Liver Hepatocellular Carcinoma; 46 LUAD, Lung Adenocarcinoma; 34 LUSC, Lung Squamous Cell Carcinoma; 86 MALY, Malignant Lymphoma; 170 MELA, Melanoma; 48 OV, Ovarian Adenocarcinoma; 70 PRAD, Prostate Adenocarcinoma; 37 SARC, Sarcoma; 25 STAD, Stomach Adenocarcinoma; 12 THCA, Thyroid Carcinoma; 34 UCEC, Uterine Corpus Endometrial Carcinoma). (e) Fraction of rearrangement breakends in tumor suppressor genes and oncogenes that are not fully resolved by SRS. Source data

To study the sequence context around loose ends, we defined a canonical axis originating at the loose end with coordinates increasing along the DNA strand whose 3′ terminus matches the side of a segment on which a loose end is found, which we refer to as the loose end’s ‘forward’ strand (Fig. 2a). We next asked whether loose ends occurred preferentially at reference sequence repeats. Indeed, we found that unmappable bases were enriched near loose ends, most frequently LINE elements (Fig. 2b and Extended Data Fig. 5a). We next reasoned that some loose ends would result from the somatic fusion of mappable bases to unalignable sequences. Confirming this, we found a tumor-specific enrichment of repetitive and foreign sequences, including satellite and viral sequences, mated to reads on the forward (but not reverse) strand of somatic loose ends (Fig. 2c and Extended Data Fig. 5b).

To identify distinct classes of repetitive SVs missing from SRS whole-genome profiles, we systematically classified tumor-specific sequences fused to each somatic loose end through assembly or consensus alignment (Fig. 2d and Methods). Overall, 55% of somatic loose ends showed evidence of tumor-specific fusion to a distal sequence. For over half of these (33% of somatic loose ends), the distal sequence aligned uniquely, indicating that these were fully mapped breakends missed by the initial junction caller (Fig. 2e) (SvAbA^18^). In 23% of somatic loose ends (3% of detected breakends), the distal sequence was repetitive or foreign and could not be unambiguously placed on any reference (ambiguously mapped breakends; Fig. 2e). Finally, 45% of somatic loose ends (6% of detected breakends) did not map to any distal location (partially mapped breakends; Fig. 2e). Notably, partially mapped breakends were enriched in boundaries of large (>1-Mb) CN-unmappable regions (odds ratio (OR) = 3.8; *P* < 2 × 10^−16^) (Extended Data Fig. 5c), indicating that some represented CN changes shifted away from a CN-unmappable SV breakend (for example, centromeric breakends causing arm-level chromosomal changes).

Combining fully mapped breakends across both loose ends and junctions indicated that 91% of JaBbA v1 breakends could be uniquely mapped. Notably, the fraction of partially or ambiguously mapped breakends did not vary substantially across cancer types (Extended Data Fig. 5d; range of 5–33%) or established cancer drivers (Extended Data Fig. 5e; range of 0–38%), although we observed tumor types (for example, acute myeloid leukemia) and cancer genes (*SMARCB1*, *TSC2* and *FGFR3*) with higher (>25%) fractional burdens. Given the estimated recall of JaBbA v1 (~96%), these results suggest that 87% of cancer SVs in the 87% of the genome that is CN-mappable can be fully resolved by SRS.

### Long-molecule validation

**Extended Data Fig. 6:**
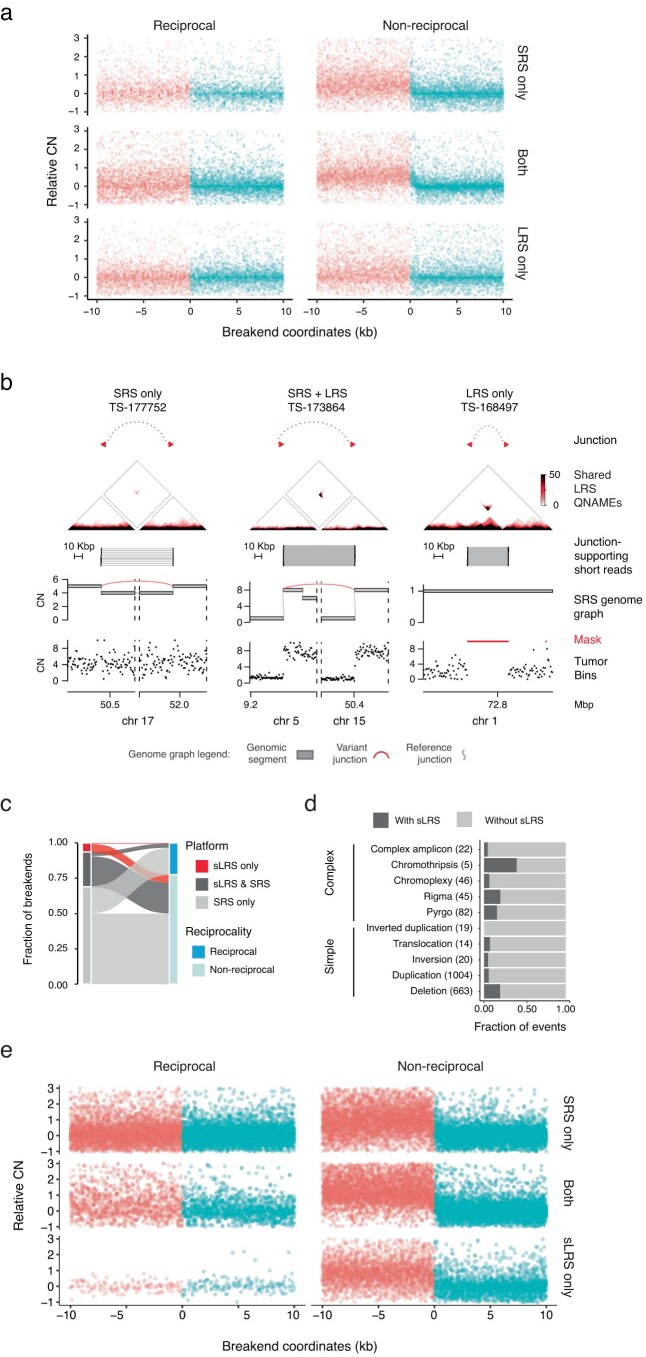
Comparison of breakends detected by SRS and either LRS or sLRS. (a) CN changes around breakends (JaBbA v1 loose ends and junction breakends) across reciprocal (left) and non-reciprocal (right) breakends detected by SRS and/or LRS (see Fig. 2 legend and Methods for more details). Bins are plotted in breakend coordinates (similar to loose end coordinates, oriented to the forward strand of the loose end or junction breakend) and normalized so that the relative CN of the segment harboring the breakend is 0. (b) Example of junctions called by SRS and LRS. Tracks from top to bottom show shared qnames per pair of genomic bins, SRS read pairs supporting the variant junction, the SRS-only genome graph, and the tumor read depth. The first example shows a call made by only short reads, the next shows a call made by both LRS and SRS, the rightmost panel shows calls made by only LRS. Of note, the rightmost deletion is missed by JaBbA as it spans a CN-unmappable region (red). (c) Alluvial plot showing high-confidence breakends called by either sLRS, SRS, or by both platforms, and whether the breakend is reciprocal or non-reciprocal. sLRS breakends are taken from tumor-specific junctions found by at least two of the three sLRS SV callers (LinkedSV^57^, GROC-SV^58^, and NAIBR^59^). SRS breakends comprise junction breakends and loose ends in the JaBbA v1 genome graph. (d) Fraction of complex and simple SV calls made using sLRS and SRS junctions or SRS junctions only. (e) CN changes around reciprocal (left) and non-reciprocal (right) breakends detected by SRS and/or sLRS, similar to (a). Source data

To orthogonally assess these SRS-derived estimates of missing somatic SVs, we profiled the whole genomes of 11 melanoma (*n* = 10) and breast cancer (*n* = 1) tumor samples and their matched normal tissues with both SRS and Oxford Nanopore Technologies long-read sequencing (LRS; median read N50 of 11 kb; median coverage of 73× and 32× for tumor and normal samples, respectively). After calling large (>10-kb) somatic SVs in CN-mappable regions (Methods), we found a strong overlap (87%, 7,258 breakends) between LRS and SRS breakends, including 77% overlap with fully mapped SRS breakends (Fig. 2f). The majority of junction calls identified by either platform had local read depth changes that were consistent with breakend topology; reciprocal breakends were copy-neutral, whereas nonreciprocal breakends showed a CN drop along their forward strand (Extended Data Fig. 6a). This analysis along with manual inspection of long and short read support at inidivudal junctions (Extended Data Fig. 6b) suggested that both SRS-only and LRS-only junctions comprise largely true positives; combining SRS and LRS breakend counts suggests that SRS missed ~12% of breakends. This result is consistent with our simulation-based estimate of recall (Fig. 1b and Extended Data Fig. 3f). Notably, we found a similar proportion of reciprocal and non-reciprocal breakends among those detected and missed by SRS (Fig. 2f), indicating that reciprocal and copy-neutral breakends do not comprise the bulk of missed structural variation in cancer genomes. These results confirm our SRS findings that most cancer SVs are nonreciprocal and copy-altered (Fig. 1e).

We next asked whether LRS improved SV event detection, which relies on the recognition of high-order patterns across multiple junctions^3,4^. Although LRS did not help identify many additional simple or complex events relative to SRS (Fig. 2g), LRS junctions also resolved breakends at complex SVs found by SRS, including for chromothripsis, pyrgo, rigma and templated insertion chains^3,4^. The incorporation of LRS junctions enabled more complete haplotype reconstruction at loci where SRS found loose ends (Fig. 2h).

As additional validation of our results, we analyzed 27 high-purity (purity of >0.5) breast cancer and matched normal samples with both SRS and synthetic LRS (sLRS) whole-genome profiles (10x Genomics linked reads, median N50 molecule length of 23 kb, median coverage of 173× and 98× in tumor and normal samples, respectively; Methods)^19^. Similar to LRS, most sLRS SV calls (Methods) overlapped with SRS breakends, showed concordant patterns of reciprocality and CN change, and yielded similar complex SV calls in sLRS junction-augmented genome graphs (Extended Data Fig. 6c–e). These breast cancer and melanoma LRS and sLRS results are consistent with our pan-cancer finding that SRS captures most large cancer SVs in CN-mappable regions.

### Loose ends reveal neotelomeres

**Fig. 3:**
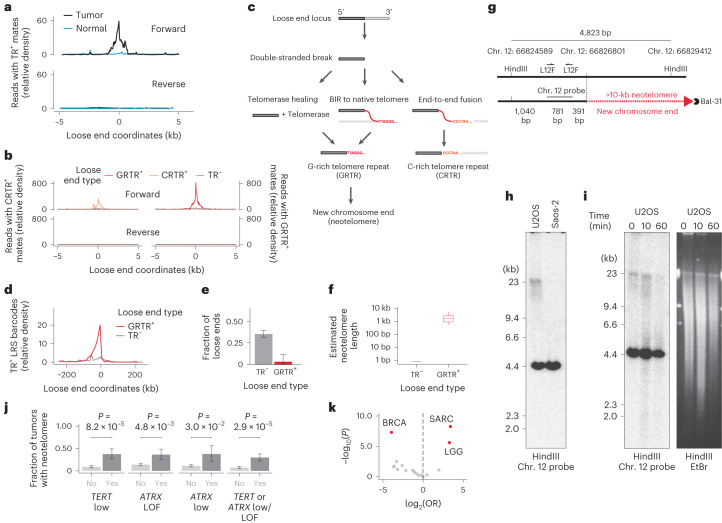
Loose ends reveal neotelomeres. **a**, Density of reads mated to telomere repeats near loose ends. TR, telomere repeat. **b**, Density of reads mated to GRTRs and CRTRs on the forward and reverse strands of GRTR^+^, CRTR^+^ and telomere repeat-negative loose ends. **c**, Potential etiologies of telomere repeats fused to loose ends. **d**, Density of sLRS barcodes harboring telomere repeat-positive read pairs near GRTR^+^ loose ends relative to telomere repeat-negative loose ends. Telomere repeat-positive read pairs are defined as a read pair in which one mate is spanned entirely by G-rich telomere repeats and the other by C-rich telomere repeats. **e**, Fraction of loose ends fused to a unique distal interstitial location via sLRS (Methods). Data are shown as mean ± 95% confidence interval (CI) (*n* = 71 GRTR^+^ loose ends and 28 CRTR^+^ loose ends from 14 tumor samples). **f**, Estimated telomere length at GRTR^+^ loose ends compared to telomere repeat-negative loose ends (*n* = 71 GRTR^+^ loose ends from 14 tumor samples). In box plots, the line represents the median, the body represents the IQR and whiskers extend to 1.5 times the IQR. **g**, Schematic of the neotelomere detection assay. **h**, Southern blot showing a diffuse ~23-kb band in U2OS cells but not in a control cell line (Saos-2). Both cell lines show the 4.4-kb HindIII control band. **i**, The U2OS-specific band disappears after exonuclease (Bal-31) digestion of genomic DNA before HindIII digestion (left) without altering the overall size distribution of DNA fragments (right). EtBr, ethidium bromide. Time refers to length of Bal-31 exposure. For **h**,**i**, the experiment was repeated four times with similar results. Panels show uncropped images of the entire gel lanes. **j**, Fraction of tumors with a neotelomere (that is, GRTR^+^ loose end) across the given categories. ‘*ATRX* low’ corresponds to *ATRX* RPKM of <500 and ‘*TERT* low’ corresponds to *TERT* RPKM of 0. Error bars are the 95% CIs on the binomial proportion. LOF, loss of function. **k**, Tumor type enrichment of GRTR^+^ loose ends. Tumor types with a statistically significant association with GRTR^+^ loose end burden are highlighted in red. See the Fig. 1d legend for definitions of the abbreviations. In **j**,**k**, *P* values were calculated by two-sided Wald’s test on the coefficients of a negative binomial generalized linear model (Methods). In **k**, values with ∣log(OR)∣ > log(1.5) and false discovery rate (FDR) < 0.1 after Benjamini–Hochberg correction are highlighted in red. Source data

We next sought to investigate specific mutational processes engendering loose ends. We observed that a fraction (4.8%) of ambiguously mapped loose ends (0.01% of all breakends) were fused to telomere repeats, as evidenced by telomere repeat-positive sequences mated to reads on the positive loose end strand (Fig. 3a). We refer to these breakends as telomere repeat-positive loose ends and surmised that they might represent neotelomeres, telomere-stabilized chromosome ends at previously interstitial genomic loci.

Telomere repeat-positive mates were found on the forward strand of telomere repeat-positive loose ends, but not on the reverse strand or in matched normal samples (Fig. 3a), indicating that these were neither telomere insertions^20,21^ nor constitutional neotelomeres^22,23^. Deeper analysis of telomere repeats at loose ends revealed strong strand bias, with loose ends harboring either G-rich (GRTR) or C-rich (CRTR) repeats but not both (Fig. 3b). The GRTR pattern is consistent with a neotelomere, whereas the CRTR pattern is consistent with the fusion of an interstitial sequence to a native chromosome end (Fig. 3c, right). The predominance of the GRTR pattern among telomere repeat-positive loose ends, in combination with the tumor specificity and forward strand bias, suggested that somatic neotelomeres are frequent in cancer.

To better assess sequences fused to GRTR^+^ loose ends, we profiled three cancer cell lines (U2OS, NCI-H526 and NCI-H838) with sLRS (Methods). We found telomere repeat-positive linked reads within 5 kb of 26 of 31 GRTR^+^ loose ends (83.8%) (Methods). Telomere repeat-positive linked reads were found up to 50 kb upstream of each GRTR^+^ loose end, indicating power to map distal fusion partners at these loci (Fig. 3d). In contrast to sLRS junctions and telomere repeat-negative loose ends, linked reads at GRTR^+^ loose ends rarely (<1.5%) mapped to distant chromosomal locations, consistent with new chromosome ends (Fig. 3e). Quantitative analysis of repeat counts at linked reads mapping to these loci (Methods) revealed 2.4 ± 1.3 (s.d.) kb of telomere repeats per GRTR^+^ locus, in line with previous estimates of native cancer telomere lengths^20^ (Fig. 3f).

**Extended Data Fig. 7:**
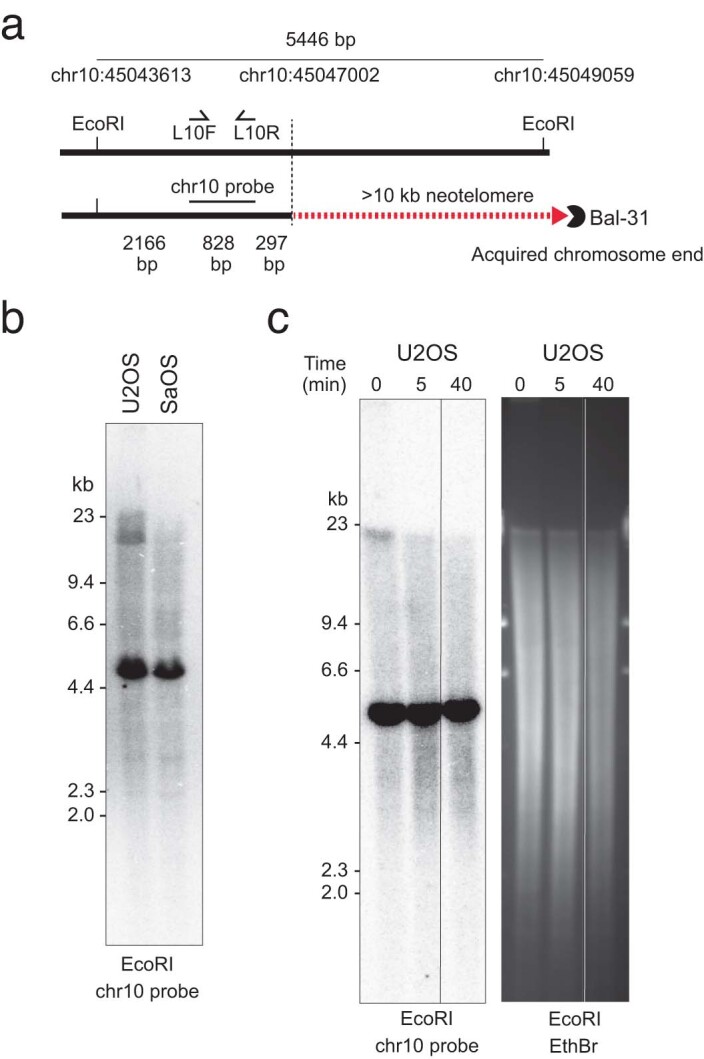
U2OS neotelomere validation. (a) Schematic of neotelomere detection assay. Genomic DNA was subjected to EcoRI digestion followed by Southern blotting with a radiolabeled probe to the site of a chr 10 GRTR+ loose end (bottom track). Alleles that lack a neotelomere at this location will show a focal ≈ 5 kbp control band. A neotelomere will yield a diffuse band that is sensitive to digestion by the exonuclease Bal-31 prior to EcoRI digestion. (b) A Southern blot shows a diffuse high molecular weight band in U2OS but not in a control cell line (SAOS-2), consistent with a neotelomere. Both cell lines show the ≈ 5 kbp EcoRI control band. (c) The U2OS-specific band disappears when double stranded DNA ends of intact genomic DNA are digested by Bal-31 for over 5 minutes prior to EcoRI digestion and probe hybridization, confirming a new chromosome end (left). Bal-31 digestion does not substantially alter the overall size distribution of ethidium bromide (EthBr) labeled DNA fragments in the EcoRI digest (right). Time refers to length of Bal-31 exposure. Experiment repeated three times with similar results. Panels show uncropped images of entire gel lanes.

To confirm that GRTR^+^ loose ends were indeed chromosome ends, we performed Southern blot analysis on restriction-digested U2OS and control (Saos-2) genomic DNA using radiolabeled probes against two U2OS GRTR^+^ loose ends. At each locus (Fig. 3g and Extended Data Fig. 7a), we found a small (<5-kb) band consistent with an unaltered reference allele and a longer U2OS-specific diffuse band consistent with a neotelomere (Fig. 3h and Extended Data Fig. 7b). To further investigate the nature of these nonreference bands, we subjected intact genomic DNA to exonuclease (Bal-31) digestion^24^. The U2OS-specific (but not wild-type) bands disappeared with prolonged exonuclease exposure (Fig. 3i and Extended Data Fig. 7c), consistent with their origin at a chromosome end. These results establish these two U2OS GRTR^+^ loose ends as bona fide neotelomeres.

We next hypothesized that telomerase-mediated healing of double-stranded DNA breaks might give rise to neotelomeres (Fig. 3c, left)^25^. However, neotelomeres were not found more frequently in tumors that amplified *TERT* or expressed it at high levels (CN > 2 ploidy, expression *z* score > 2). Instead, neotelomeres were enriched in samples with low or negligible *TERT* expression (reads per kilobase per million mapped reads (RPKM) = 0) (Fig. 3j). Tumors that lack telomerase may activate the alternative lengthening of telomeres (ALT) pathway, a break-induced replication (BIR) process (Fig. 3c, middle) suppressed by ATRX^26^. Indeed, we found that neotelomeres were significantly more common in tumors harboring truncating mutations in *ATRX* than in *ATRX*-wild-type cancers (Fig. 3j). Furthermore, we found that several ALT-associated cancers, including sarcomas (18%; OR = 6.47; *P* = 1.95 × 10^−5^) and low-grade gliomas (12.3%; OR = 3.92; *P* = 4.1 × 10^−3^), had the highest rate of GRTR^+^ loose ends relative to other tumor types (Fig. 3k). These results indicate that GRTR^+^ loose ends and neotelomeres may be a new hallmark of the ALT phenotype.

### Loose ends link viral integration to amplicon formation

**Extended Data Fig. 8:**
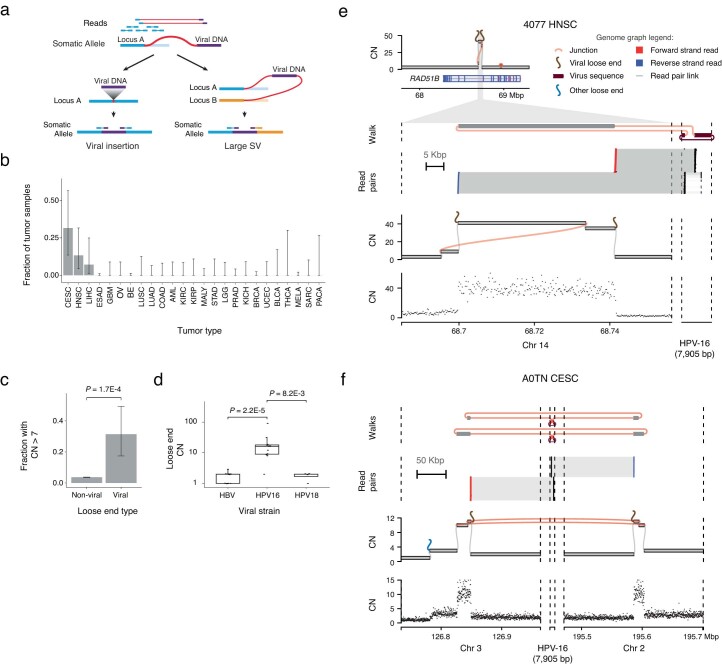
Viral loose ends are amplified in virus-driven cancers. (a) Schematic contrasting viral loose ends and insertions. Virus integrations causing SVs will give rise a tumor-specific viral contig associated with a pair of distant loose ends (right) while virus insertions arise from viral integration into unrearranged DNA (left). (b) Bar plot showing fraction of samples containing at least one viral loose end by tumor type. Error bars show mean fraction ± 95% confidence interval. Only tumor types with ≥10 samples are shown (n = 48 AML, 20 BLCA, 127 BRCA, 15 CESC, 57 COAD, 186 ESAD, 43 GBM, 30 HNSC, 40 KICH, 24 KIRC, 19 KIRP, 41 LGG, 28 LIHC, 46 LUAD, 34 LUSC, 86 MALY, 170 MELA, 48 OV, 70 PRAD, 37 SARC, 25 STAD, 12 THCA, 34 UCEC). See Extended Data Fig. 5d caption for expanded tumor type abbreviations. (c) Association between viral loose ends and high-CN amplicons. Left: Viral loose ends are more likely to have high CN (≥ 7) compared to all other loose ends (n = 32 viral loose ends, 68354 other loose ends). Error bars show mean fraction ± 95% confidence interval. (d) CN of viral loose ends separated by virus type. Box plots: line (median), body (IQR), whiskers (1.5 times IQR). *P*-values calculated with two-sided Wilcoxon rank sum test. (e-f) Example viral loose ends from TCGA HNSC sample 4077 (e) and CESC sample A0TN (f). From top, “walk" representation of proposed circular allele; alignments of read pairs with discordant alignments between human and viral genome; JaBbA v1 genome graph showing two loose ends corresponding to the discordant read alignments; binned purity / ploidy transformed read depth. Genome graph legend in panel e applies to both e and f. Source data

Surveying additional mutational processes engendering loose ends, we found ambiguously mapped somatic breakends fused to viral sequences, indicating junctional viral integration at large SVs (Extended Data Fig. 8a). While the integration of viral sequences into otherwise unrearranged loci (Extended Data Fig. 8a, left) has been widely studied in cancer^27,28^, the role of viruses in causing chromosomal-scale SVs (Extended Data Fig. 8a, right) has been a topic of only recent interest^29–31^. Somatic loose ends harboring tumor-specific viral sequence (viral loose ends) were rare overall (~1% of cancers), although enriched in cancer types with viral etiology in our dataset^4^: cervical squamous cell carcinoma (CESC; 32%), liver hepatocellular carcinoma (LIHC; 13%) and head and neck squamous cell carcinoma (HNSC; 7%) (Extended Data Fig. 8b). Consistent with previously characterized viral integration patterns, we found viral loose ends fused to oncogenic HPV sequences in CESC and HNSC and hepatitis B virus (HBV) sequences in LIHC^27^.

Breakends initiating complex amplifications are themselves likely to be amplified^4^. Viral loose ends were frequently amplified (CN > 7) relative to nonviral loose ends (*P* = 1.7 × 10^−4^; OR = 8.66) (Extended Data Fig. 8c), and HPV-16 loose ends had higher mean CN than either HPV-18 or HBV loose ends (*P* = 8.2 × 10^−3^ and *P* = 2.2 × 10^−5^, respectively, Extended Data Fig. 8d). Among these was an HNSC tumor (TCGA-4077) locus where two high-copy viral loose ends on chromosome 14 flanking an intronic region of the *RAD51B* gene were fused to opposite ends of the HPV-16 genome (Extended Data Fig. 8e). This locus is consistent with an ecDNA where HPV-16 is fused between two ends of a long-range duplication junction. This and other similar amplicon structures with high-copy viral loose ends (Extended Data Fig. 8e,f) point to HPV-16 integration as an initiating event in SV evolution, rather than a viral insertion into an existing ecDNA.

### Crossover between parental homologs is rare in cancer

**Fig. 4:**
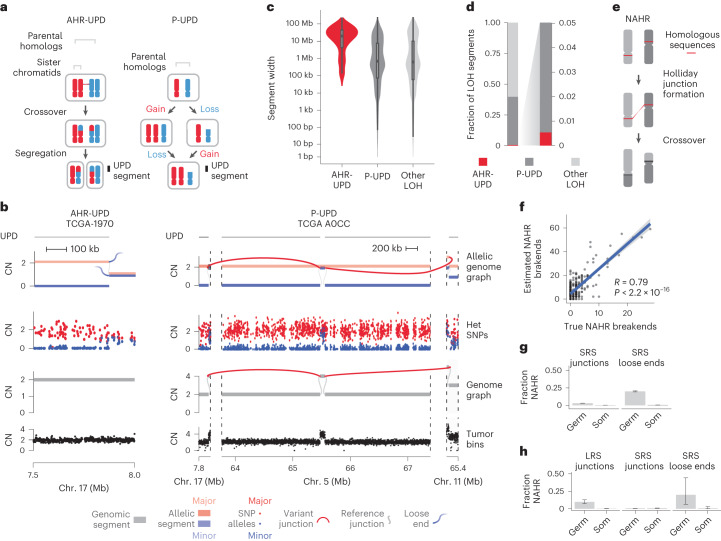
AHR rarely drives CN-mappable breakends. **a**, Schematic showing mechanistic differences between AHR and P-UPD, two mechanisms that give rise to segmental UPD. **b**, Examples of AHR-UPD (left) and P-UPD (right). The allelic graph (top subpanel) shows parental homolog-specific CN, which matches purity- and ploidy-transformed allelic SNP read counts (scatterplot, second subpanel from top) (Supplementary Information). The AHR-UPD locus shows no breakends in the total CN JaBbA v1 graph (third subpanel) but a pair of loose ends in the allelic graph. By contrast, the P-UPD locus does not harbor a pair of allelic graph loose ends, but rather contains a copy-altered breakend in both the allelic and total CN JaBbA v1 graphs. Het, heterozygous. **c**, Width distribution of segments produced by AHR-UPD, P-UPD and all other LOH (*n* = 545 AHR-UPD ranges, 39,877 P-UPD ranges and 61,469 other LOH ranges from 1,330 tumors). In box plots, the line represents the median, the body represents the IQR and whiskers extend to 1.5 times the IQR. **d**, Fractional contribution of P-UPD, AHR-UPD and other forms of LOH to the total number of LOH segments. **e**, Schematic of NAHR. **f**, Number of estimated (*y* axis) versus true (*x* axis) NAHR-mediated breakends per simulated sample (*n* = 500 simulated genomes). The blue line shows the line of best fit, with Pearson’s correlation coefficient provided on the graph; error bands show the standard error of the prediction. The *P* value was calculated from the *t* distribution of Pearson’s correlation coefficient test statistic. **g**, Fraction of somatic junctions, somatic loose ends and germline loose ends consistent with NAHR rearrangements in the SRS pan-cancer whole-genome cohort (*n* = 1,330 samples). Error bars represent the 95% CIs on the binomial proportion. Germ, germline; Som, somatic. **h**, Fraction of germline and somatic LRS junctions, SRS junctions and SRS loose ends consistent with NAHR in a separate melanoma and breast cancer cohort with paired SRS and LRS whole-genome profiles (*n* = 11 samples). Error bars represent 95% CIs on the binomial proportion. Source data

We next asked whether loose ends could be used to assess the contribution of aberrant homologous recombination to cancer rearrangements. Homologous recombination-driven crossover between parental homologs (allelic homologous recombination, or AHR) is a hallmark of meiosis^32^. Although AHR has been observed in somatic cells^33^, its contribution to cancer structural variation is unclear. AHR crossovers lead to segmental uniparental disomy (UPD) in approximately half of segregants (Fig. 4a, left). In balanced allelic graphs, AHR crossovers manifest as reciprocal pairs of partially mapped and copy-neutral loose ends on distinct parental homologs (Fig. 4b, left, and Methods). Notably, this form of UPD (AHR-UPD) is mechanistically distinct from UPD arising through progressive acquisition of nonhomologous recombination (for example, end joining)-driven rearrangements and/or chromosomal missegregation (progressive UPD, or P-UPD; Fig. 4a,b, right).

**Extended Data Fig. 9:**
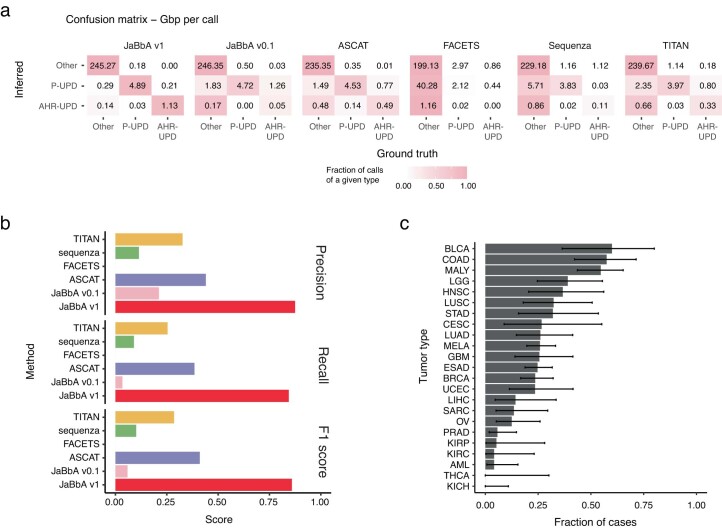
Benchmarking AHR detection. (a) Confusion matrix comparing AHR-UPD and P-UPD labels assigned using the allele-specific CN profile produced by various CN inference algorithms on a simulated dataset of 200 samples. AHR-UPD calls were defined as segments with major allele CN = 2 and minor allele CN = 0, immediately adjacent to a heterozygous segment with total CN = 2. The remaining segments with major allele CN = 2 and minor allele CN = 0 are annotated as P-UPD. The calls made by each CN inference algorithm are shown as rows, while the ground truth call is shown as columns. The number in each box represents the Gbp of each call across all simulated samples, while the color scale is normalized by row (representing precision). Only segments with width > 10 kbp were considered in this analysis. (b) Overall precision, recall, and F1 score of AHR calls made by each algorithm. (c) Prevalence of AHR in each cancer type. Only tumor types with ≥10 cases are included. (n = 48 AML, 20 BLCA, 127 BRCA, 15 CESC, 57 COAD, 186 ESAD, 43 GBM, 30 HNSC, 40 KICH, 24 KIRC, 19 KIRP, 41 LGG, 28 LIHC, 46 LUAD, 34 LUSC, 86 MALY, 170 MELA, 48 OV, 70 PRAD, 37 SARC, 25 STAD, 12 THCA, 34 UCEC). See Extended Data Fig. 5d caption for expanded tumor type abbreviations. Error bars show mean fraction per tumor type ± 95% confidence interval. Source data

In our simulations (Extended Data Fig. 3a and Methods), JaBbA v1 distinguished AHR-UPD from P-UPD with both high precision (84.4%) and high recall (87.4%), substantially outperforming previous allelic CN algorithms (with precision ranging from 11–44%) (Extended Data Fig. 9a,b). Analysis of segment width distributions showed that AHR-UPD was distinct from P-UPD, whose distribution closely mirrored that of other forms of loss of heterozygosity (LOH; Fig. 4c). Likewise, AHR-UPD events were large (median width of 19.8 Mb), unlike P-UPD events (median width of 0.69 Mb) and other forms of LOH (median width of 0.62 Mb), which were focal (Fig. 4c).

Although AHR was found in many cancers (24% of all tumors) and specific tumor types (for example, 55% of cases of malignant lymphoma) (Extended Data Fig. 9c), it contributed to a minority of UPD events, most of which were progressive (31% P-UPD versus 1% AHR-UPD by total width) (Fig. 4d). Overall, a small minority of detected cancer breakends (<1%) arose by AHR (including non-UPD LOH). On the basis of an approximate rate of 0.5 AHR events per tumor and 100 cell divisions in the average ancestral cancer clone, and barring effects of selection, we estimate a rate of 10^−12^ AHR events per base pair per cell division. This is four orders of magnitude lower than the rate of meiotic recombination in human gametes, suggesting that AHR events are infrequent in somatic evolution^34^.

### Germline but not somatic loose ends are consistent with NAHR

**Extended Data Fig. 10:**
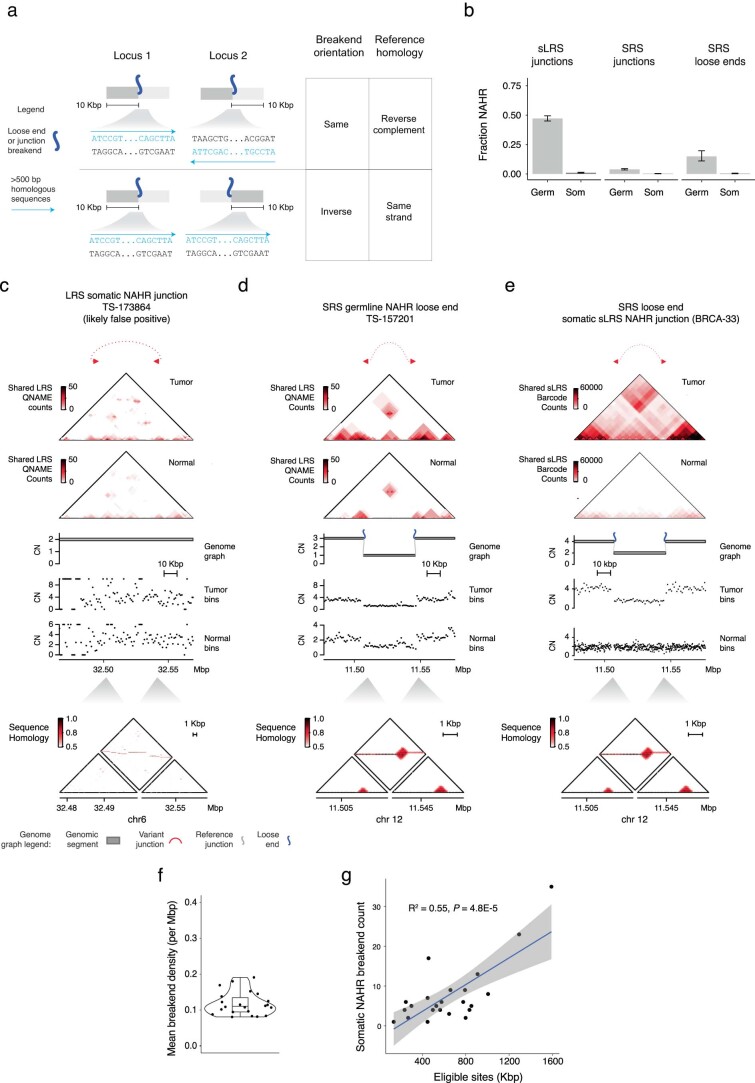
Detecting NAHR in SRS, LRS, and sLRS whole genome profiles. (a) Schematic of method for detecting NAHR at SRS junction or loose end associated breakends. Locus 1 and 2 are sites of breakends (blue lines) either belonging to a junction or a pair of loose ends in a tumor sample. A loose end was designated putative NAHR if there was at least one other loose end within the sample sharing > 500 bp of strand-specific sequence homology (>96% identity) within 10 kbp of each breakend (see Methods). Similarly, a junction was annotated as putative NAHR if > 500 bp homologous sequences were found within 10 kbp of each breakend belonging to the junction. (b) Fraction of sLRS junctions (n = 3352 somatic, 1978 germline) and SRS junctions (n = 4740 germline, 5694 somatic) and loose ends (n = 282 germline, 2397 somatic) consistent with NAHR, separated by whether the rearrangement was germline or somatic (n = 27 tumors). Error bars show mean fraction ± 95% confidence interval. Germ, germline; Som, somatic. (c) Example of a somatic LRS junction that was designated putative NAHR. Tracks from top to bottom show the number of shared reads with split alignments between pairs of loci in the tumor and matched normal LRS, purity / ploidy transformed read depth in the tumor and matched normal, the JaBbA v1 graph, and fraction of bases having exact homology. (d) Example of two germline loose ends identified as putative NAHR and validated as a germline NAHR junction in the LRS data (top heatmaps). Genome graph legend as in panel c. (e) Example of two somatic loose ends identified as putative NAHR in the sLRS data. Top two tracks show number of shared sLRS barcodes in the tumor and matched normal. Genome graph legend as in panel c. (f) Mean per-megabase density of somatic breakends on each autosome in CN-mappable regions across n = 1330 tumor genomes. Box plot elements: line (median), body (IQR), whiskers (1.5 IQR). (g) Pearson correlation between eligible homologous position pairs per chromosome and total estimated somatic NAHR breakend count per chromosome. Error bands show standard error of the prediction and *P*-value was calculated from t-distribution of Pearson’s correlation coefficient test statistic. Source data

A second mechanism by which aberrant homologous recombination can cause large SVs is through non-AHR (NAHR), or crossover between long (>500-bp) stretches of nearly identical genomic sequences at distant haploid coordinates^32,35,36^ (Fig. 4e). We reasoned that such SVs would engender pairs of loose ends with substantial (>500-bp) strand-specific sequence homology in their vicinity (Extended Data Fig. 10a and Methods)^36^. Indeed, the burden of homologous loose end pairs accurately reflected the true NAHR burden across a compendium of simulated SRS tumor whole-genome profiles (Extended Data Fig. 3a) harboring a wide range of NAHR SV fractions (1–10%) (Fig. 4f).

Analyzing breakend pairs within each tumor, we found that approximately 20% of germline loose ends (Methods) were consistent with NAHR in contrast to only about 0.5% of somatic loose ends (and 0.06% of all somatic SV breakends) (Fig. 4g). These findings are consistent with prior observations about the substantial role of NAHR in germline variation^8,37^. The somatic NAHR burden did not vary by tumor type nor was it lower in tumors harboring biallelic pathogenic mutations in DNA repair genes, including frequently mutated homologous recombination pathway mediators (*BRCA1*, *BRCA2*, *PALB2* and *RAD51C*). In summary, given a mean of 0.16 somatic NAHR events per tumor occurring across an estimated eligible territory of 2.8 × 10^8^ homologous position pairs, we estimate a somatic NAHR density of 6 × 10^−10^ events per cancer genome bp^2^ (Methods).

To validate these SRS findings in long-molecule whole-genome profiles, we analyzed 38 melanoma and breast cancer cases profiled with SRS and either LRS or sLRS. Both LRS and sLRS data confirmed our SRS findings that somatic NAHR SVs were rare (<1% of LRS junction calls) while germline NAHR SV events were common (Fig. 4h and Extended Data Fig. 10b–e). Notably, we did not identify any reciprocal somatic NAHR rearrangements, a class of SVs that may potentially be missed through analysis of SRS loose ends.

### Extrapolating beyond the CN-mappable genome

**Fig. 5:**
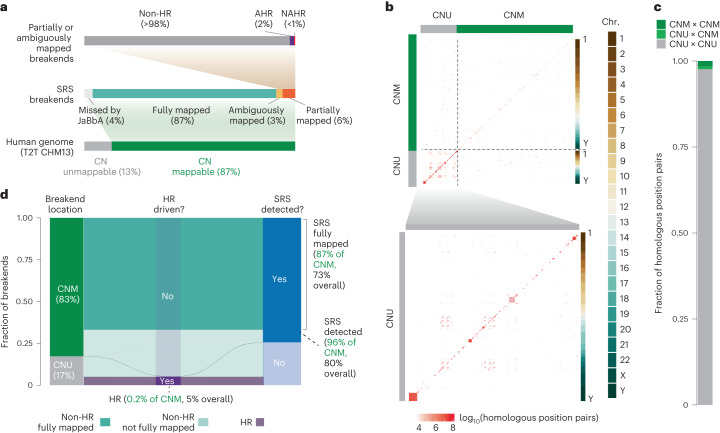
Extrapolating beyond the CN-mappable genome. **a**, Summary of SV breakends in the CN-mappable genome, including those predicted to be undetected by JaBbA v1 (Figs. 1 and 2). **b**, Heatmap showing the number of NAHR-eligible reference sequence position pairs, defined as pairs of reference positions >10 kb apart with ≥96% homology across 500 bp. The size of each bin in the genome-wide plot is 10 Mb (top subpanel) and 1 Mb (bottom subpanel, CN-unmappable zoom-in). CNU, CN-unmappable; CNM, CN-mappable. **c**, Fractional contribution of NAHR-eligible position pairs (see above) tallied across CN-unmappable and CN-mappable genome partitions. The number of position pairs with at least one site in a CN-unmappable region is expected to be ~100 times greater than the number of position pairs fully contained in CN-mappable regions. **d**, Alluvial plot showing the estimated fraction of SV breakends mapped by SRS across the genome. Colors stratify breakends on the basis of SRS mappability and homologous recombination versus other repair mechanisms. Source data

The analyses described above were limited to the 87% of the genome where CN could be reliably measured with SRS (Fig. 5a). The remaining 13% that is CN-unmappable comprises largely regions in or around telomeres and centromeres (Extended Data Fig. 2b). To assess the burden of large SVs here, we applied two simplifying assumptions: (1) the rate of NAHR between any two regions in the genome is proportional to the number of position pairs with substantial homology (>500 bp with >96% homology) between these regions and (2) the density of non-NAHR-driven rearrangements is uniform across the genome, and hence the burden of non-NAHR breakends in a given region is proportional to its width. Both of these assertions hold true, to a first approximation, across the CN-mappable genome (Extended Data Fig. 10f,g).

We used the latest telomere-to-telomere build (T2T CHM13; ref. ^38^) to estimate the number of homologous position pairs outside CN-mappable regions (Fig. 5b). We found that CN-unmappable sequences harbored ~100-fold-greater homologous position pairs (2.7 × 10^10^ bp^2^) than the CN-mappable portion of the T2T CHM13 genome build (2.8 × 10^8^ bp^2^) (Fig. 5c). This suggested that CN-unmappable regions harbor ~100 times as many NAHR SVs as CN-mappable regions. Integrating these measurements (Fig. 5a–c and Methods), we estimate that CN-mappable regions harbor 83% of all large SV cancer breakends, most of which are detected by SRS (Fig. 5d). Furthermore, even when CN-unmappable regions are taken into account, we estimate that homologous recombination contributes to a small proportion (~5%) of large cancer SV breakends (Fig. 5d).

## Discussion

As cancer whole-genome SRS efforts scale and long-molecule genome profiling technologies mature, it is important to understand the limitations of SRS, particularly for the detection of chromosomal alterations. The conventional wisdom in the field has been that SRS misses most SVs owing to the prevalence of repeats in the human genome and the unclear contribution of NAHR to somatic structural genomic evolution^8,37,39,40^. Contrary to this prevailing intuition, we find that SRS detects and maps most large (>10-kb) somatic SV breakends in CN-mappable genomic regions. Intuitively, this is because most cancer chromosomal alterations are unbalanced and nonreciprocal (Fig. 1e), thus creating a CN footprint that SRS, when guided by mass balance approaches such as JaBbA v1, can reliably detect (Fig. 1b).

Our SRS analyses suggest that long-molecule technologies (for example, LRS and sLRS) will only modestly improve the detection of chromosomal breakends. We confirm this by jointly profiling the whole genomes of cancer samples and their matched normal samples with deep long-molecule sequencing (LRS or sLRS) and SRS. Given our findings, what additional insight into SVs can long-molecule technologies hope to offer? First, long molecules will enable the phasing of junctions to nearby somatic and germline variants. Resolution of the multi-junction haplotype structure at complex SVs may substantially inform their mechanistic interpretation and functional annotation, as in a recent study from our group^19^. Second, long molecules substantially increase the sensitivity for smaller (≤10-kb) somatic SVs, which were excluded from our analyses^41–43^. Future long-molecule studies will be needed to uncover the mutational processes and selective pressures driving the evolution of these smaller SV classes, including retrotransposition events.

Our study provides some of the most definitive evidence showing that NAHR drives a small proportion (<1%) of chromosomal alterations, at least in CN-mappable genomic regions. Our NAHR estimates in the remaining 13% (Fig. 5) of the genome assume that CN-mappable and CN-unmappable regions are subject to similar mutational processes. This assertion may require re-evaluation given recent studies investigating centromeric mutational processes^44^. Other settings where homologous recombination has been invoked, such as in the recombination of extrachromosomal DNA (ecDNA)^45,46^, may similarly represent unique chromatin environments that are distinct from the remainder of the genome where homologous recombination rarely creates large SVs.

Practically, our study establishes JaBbA v1 as a state-of-the-art algorithm for cancer CN analysis, improving upon JaBbA v0.1 as well as classic ‘change point’-based CN callers (Fig. 1b). The use of mass balance in the JaBbA model provides both superior performance in detecting somatic breakends and a lens into missing cancer SVs. Our study supports the use of JaBbA v1 and, more broadly, SRS in clinical cancer cytogenetics, where whole-genome SRS is poised to become routine in an era of plummeting sequencing costs^47,48^.

## Methods

### Research compliance

The research described below complied with all relevant ethical regulations. Notably, 72 deidentified fresh-frozen samples (36 tumors and 36 matched normal tissues) were collected for SRS and LRS or sLRS profiling and analysis (see below) from patients consented to have their genomes profiled and shared at Memorial Sloan Kettering Cancer Center (MSKCC) under institutional review board approvals MSKCC 00-144, MSKCC 12-245 and MSKCC 16-675. Participants were not compensated.

### JaBbA v1 algorithm

The JaBbA v1 algorithm builds on the previous JaBbA (v0.1) algorithm introduced in Hadi et al.^4^ with two key modifications: (1) updating the JaBbA statistical model to a Laplace distribution, which improved performance and convergence, and (2) balancing allelic genome graphs across parental SNP homologs to enable breakend phasing and identification of allelic loose ends. These and other pipeline changes are visually summarized in Extended Data Figs. 1a and 2a–d. The updated algorithm is described in further detail below.

#### Genome graph structure

As previously described^4^, JaBbA infers balanced genome graphs through the solution of a mixed-integer program (MIP). A genome graph is a directed graph *G* = (*V*, *E*) whose vertices *v*_1_, *v*_2_ ∈ *V* represent strands of chromosomal segments and edges *e* = (*v*_1_, *v*_2_) ∈ *E* represent segmental adjacencies. Vertices *V* = *V*_I_ ∪ *V*_N_ comprise interstitial vertices *V*_I_ and ends *V*_N_. The ends *V*_N_ = *V*_T_ ∪ *V*_L_ further comprise reference chromosome ends *V*_T_ and loose ends *V*_L_. Edges *E* = *E*_R_ ∪ *E*_A_ ∪ *E*_L_ comprise reference edges *E*_R_, variant edges *E*_A_ and loose end edges *E*_L_, the latter of which connect each interstitial vertex to its incoming (similarly, outgoing) loose end. We use superscript notation to refer to the incoming and outgoing edges of vertices, for example, ${E}_{{\rm{L}}}^{-}(v)={E}_{{\rm{L}}}\cap {E}^{-}(v)$ and ${E}_{{\rm{L}}}^{+}(v)={E}_{{\rm{L}}}\cap {E}^{+}(v)$ to denote the (single) loose end edge that is upstream and downstream of a vertex *v*, respectively.

#### Statistical model

Given an initial genome graph, JaBbA assigns a non-negative integer CN $\kappa :\{V\cup E\}\to {\mathbb{N}}$ to every vertex and edge of *G* on the basis of (1) the principle of mass balance and (2) the likelihood of purity- and ploidy-transformed read depth data $x\in {{\mathbb{R}}}^{n}$ across *n* genomic bins. The principle of mass balance requires the CN of every vertex to be equal to the sum of its incoming edges and the sum of its outgoing edges, engendering the junction balance constraints

1 $$\kappa (v)=\mathop{\sum}\limits_{e\in {E}^{-}(v)}\kappa (e)=\mathop{\sum}\limits_{e\in {E}^{+}(v)}\kappa (e)$$

as well as a skew symmetry constraint forcing each vertex *v* and edge *e* to have the same CN as its reverse complement $\bar{v}$ and $\bar{e}$, respectively.

Each vertex *v* ∈ *V*_I_(*G*) represents a genomic segment overlapping bins *J*(*v*) ⊆ {1, …, *n*}, whose read depth *x*_*J*(*v*)_ is modeled as i.i.d. samples from a Laplace distribution with scale parameter *b*(*v*, *x*, *J*) and mean *κ*(*v*). We also apply an exponential prior with decay parameter *λ* on the count of fitted loose ends (that is, with CN > 0). Under this model, the maximum a posteriori probability estimate of *κ* minimizes

2 $$\begin{array}{rcl}f\,(G,\kappa ,x,J,\lambda )&=&\mathop{\sum}\limits_{v\in {V}_{{\rm{I}}}}\frac{| \,J(v)| }{b(v,x,J\,)}| \rho (v,x,J\,)-\kappa (v)| +\\ && \lambda \left({{\mathbb{1}}}_{\kappa ({E}_{L}^{-}(v))\ > \ 0}+{{\mathbb{1}}}_{\kappa ({E}_{{\rm{L}}}^{+}(v))\ > \ 0}\right)\end{array}$$

subject to junction balance and skew symmetry constraints. Here $\rho (v,x,J)=\frac{1}{| \,J(v)| }{\sum }_{j\in J(v)}{x}_{j}$ and the scale parameter *b* models read depth noise, which is set to $b(v,x,J)={\rm{max}}(1,\sqrt{\rho (v,x,J)})$. Finally, we allow the user to specify edges *E*_F_ ⊆ *E* (for example, high-confidence junctions) to force-incorporate into the balanced genome graph. This defines the MIP as follows:

3 $$\begin{array}{ll}\mathop{{{{\rm{minimize}}}}}\limits_{\kappa :V\cup E\to {\mathbb{N}}}&f\,(G,\kappa ,x,J,\lambda )\\ {{{\rm{subject}}}}\,{{{\rm{to}}}}&\kappa (v)=\kappa (\bar{v}),\,{\forall }_{v\in V}\\ &\kappa (e)=\kappa (\bar{e}),\,{\forall }_{e\in E}\\ &\kappa (v)=\mathop{\sum}\limits_{e\in {E}^{-}(v)}\kappa (e)=\mathop{\sum}\limits_{e\in {E}^{+}(v)}\kappa (e),{\forall }_{v\in {V}_{I}}\\ &\kappa (e) > 0,\,{\forall }_{e\in {E}_{F}}\end{array}$$

The solution of equation (3) yields a balanced genome graph (*G*, *κ*), which minimizes the number of loose ends used (that is, with CN > 0) while maximizing the likelihood of the read depth data *x*. The use of a Laplace instead of a Gaussian distribution in the likelihood allows the solution of a linear rather than a quadratic MIP, substantially improving scale and convergence relative to the previous formulation^4^. See Supplementary Note 1 for a full derivation.

#### Allelic mass balance

We extended JaBbA to use mass balance in the inference of allele-specific CN. To do so, we generate an allelic genome graph $\hat{G}$ from the original (total CN) balanced genome graph (*G*, *κ*), where every vertex in *G* gives rise to two allelic vertices in $\hat{G}$ and every edge in *G* gives rise to four allelic edges in $\hat{G}$. In addition to junction balance (equation (1)) and skew symmetry (equation (3)) constraints, we constrain the CN of allelic vertices mapping to a given vertex *v* in *G* to sum to that vertex’s total CN *κ*(*v*) (similarly for edges). We additionally allow at most one of the four variant edges in $\hat{G}$ that arise from the same variant edge in *G* to have nonzero CN. We also allow at most one of the two incoming (similarly, outgoing) reference edges associated with an allelic node in $\hat{G}$ to have nonzero CN. The latter two constraints apply the ‘infinite sites’ assumption, which states that each variant could have occurred only once in evolution (and hence on a single parental homolog). To balance the allelic genome graph, we solve a MIP that identifies the allelic vertex and edge CN assignment that maximizes the probability of allelic counts subject to these constraints. Full details of allelic mass balance are provided in Supplementary Note 1.

### JaBbA v1 pipeline

In addition to algorithmic improvements, the JaBbA v1 pipeline includes additional data processing improvements compared to the previous version^4^: (1) correction of sample-specific bias in tumor read depth and (2) rigorous definition of CN-mappable regions. Unlike previously^4^, 1-kb binned read depth *x* is obtained via dryclean^53^, a robust principal-components analysis-based algorithm to remove systematic low-rank biases in binned read depth using a panel of normal samples (Supplementary Note 2). In addition, we mask bins that occur in CN-unmappable regions of the genome (see below for details) and use purity and ploidy estimates to transform read depth into CN units (Supplementary Note 2). The JaBbA v1 pipeline then applies CBS^54^ to *x* and takes the union of the resulting segment endpoints with SvAbA^22^ junction breakends to construct a preliminary genome graph.

In practice, we use three iterations of total CN MIP optimization (equation (3)) followed by allelic mass balance. After each total CN iteration, the results are processed to yield a simplified graph where reference adjacent segments are merged if a loose end or variant junction with CN > 0 does not exist at their interface. The first MIP iteration takes as input only large (>10-kb) and high-confidence (FILTER = PASS) SvAbA junctions and CBS segment breakends. Clusters of high-confidence reciprocal SVs are constrained into the model (that is, by including in the set *E*_F_ in equation (3)). The second MIP iteration augments the graph from the first iteration with low-confidence SvAbA junctions located within 10 kb of fitted loose ends. The final MIP iteration refits the graph from the second iteration but adds a noninteger chromosome-specific offset that prevents hypersegmentation from small inaccuracies in purity and ploidy estimation or subclonal CN changes. Allelic mass balance is then run on the balanced genome graph output of the final MIP iteration. To optimize AHR detection, before allelic mass balance, large (>1-Mb) segments of the balanced genome graph are further split by running CBS on minor allelic count vectors (see Supplementary Note 2 for details of chromosomal bias correction and AHR detection).

### Mappability analysis

We performed exhaustive self-alignment of all 101-mers in the GRCh37 reference to identify base-unmappable positions, that is, those whose corresponding 101-mer gave rise to at least one full-length supplementary alignment or harbored at least one masked (N) base. A position was then called CN-unmappable if more than 90% of the bases in a 1-kb window around that position were base-unmappable; otherwise, it was called CN-mappable. An analogous approach was used to determine GRCh38 and 150-bp mappability (Extended Data Fig. 2d). Additional details are provided in Supplementary Note 2.

### Short-read whole-genome sequencing

SRS whole-genome profiles for 1,330 high-purity (>0.5) tumors (inferred sex: 586 female, 744 male; age: 4–90 years, 164 unknown) and 326 cell lines (provided sex: 139 female, 170 male, 17 unknown; age: 0.25–74.05 years, 46 unknown) were obtained from a previous study published by Hadi et al.^4^. Additional SRS whole-genome libraries were prepared using the Illumina TruSeq DNA PCR-free Library Preparation Kit and profiled on an Illumina NovaSeq 6000 sequencer with 2 × 150-bp cycles. Following GRCh37 alignment, data processing and standard whole-genome variant calling, we ran the JaBbA v1 pipeline (see above) and previously published somatic CN callers (JaBbA v0.1 (ref. ^4^), ASCAT v2.5.2 (ref. ^14^), FACETS v0.6.2 (ref. ^17^), Sequenza v3.0.0 (ref. ^16^) and TITAN v1.28 (ref. ^15^)). Additional details regarding SRS library preparation, data processing and variant calling are provided in Supplementary Note 3.

### Long-read whole-genome sequencing

LRS profiles were generated for ten melanomas and one triple-negative breast cancer collected at MSKCC (collection details above; inferred sex: six female, five male; age: >17 years). Following high-molecular-weight (HMW) DNA extraction, LRS was performed on the Oxford Nanopore Technologies PromethION sequencer using R10 chemistry with two flow cells per tumor and one flow cell per normal sample. Following GRCh37 long-read alignment (minimap2 v2.17), LRS SV junction calls were identified from the two-way consensus of four callers: cuteSV (release v2.0.2; ref. ^50^), SAVANA (release 0.2.3; ref. ^52^), SVIM (release 2.0.0; ref. ^49^) and Sniffles2 (release v2.0.7; ref. ^51^). Callers were run on tumor and normal samples separately (cuteSV, SVIM, Sniffles2) or in paired mode (SAVANA). Tumor and normal junction calls with identical orientation and 1-kb padded overlap were merged across algorithms. Additional details regarding LRS library preparation and data processing are provided in Supplementary Note 4.

### Synthetic long-read whole-genome sequencing

sLRS whole-genome profiling was performed on 25 breast cancer tumor–normal pairs (collection details above; inferred sex: 25 female, 0 male; age: >17 years) and a panel of 8 ATCC cell lines (provided sex: 5 male, 3 female; age: unknown) previously profiled with SRS by the Cancer Cell Line Encyclopedia^55^. In brief, HMW DNA was subjected to 10x Genomics Chromium Genome library preparation and sequenced on an Illumina NovaSeq 6000 sequencing system to approximately 30× base and 170× physical coverage. 10x Genomics linked reads were aligned to GRCh37 using the EMerAld aligner (v0.6.2)^56^. To nominate SV junctions, we applied a consensus of three algorithms (LinkedSV, https://github.com/WGLab/LinkedSV (commit 1b77a14)^57^; GROC-SV, https://github.com/grocsvs/grocsvs (v0.2.6)^58^; NAIBR, https://github.com/raphael-group/NAIBR (commit 15eba96)^59^) run on tumor and normal sLRS alignments. Tumor and normal junction calls with identical orientation and 1-kb padded overlap were merged across algorithms. Somatic SVs were then called as junctions found in tumors by two or more algorithms and undetected in the normal sample. Additional details regarding sLRS profiling and data processing are provided in Supplementary Note 4.

### Short- versus long-read platform comparisons

We used 1-kb strand-specific overlap of SRS breakends (junctions and loose ends) and LRS/sLRS junction breakends to assess concordance between SV calling platforms. To assess the ability of LRS or sLRS junctions to resolve SRS loose ends, we applied an additional iteration of junction balance to SRS-derived balanced genome graphs, including additional LRS or sLRS junctions as input. We then overlapped loose ends in the original SRS genome graph with junctions incorporated into the LRS/sLRS genome graph. If a loose end was within 1 kb of an LRS breakend or within 10 kb of an sLRS junction breakend on the same strand, we considered that loose end to have been resolved by LRS/sLRS. We applied gGnome^4^ to annotate and compare complex SV events across SRS, LRS and sLRS JaBbA graphs and used genomic overlaps to identify shared versus platform-specific calls.

### Loose end classification

To identify candidate distal mappings for loose ends, we used Fermi^60^ local assembly (https://github.com/mskilab-org/RSeqLib) and realignment of loose end-associated reads and mates. Fermi contigs were assessed for tumor and normal read support through BWA realignment of reads to contigs and the reference (https://github.com/mskilab-org/readsupport) to uncover tumor-specific contigs with distal alignments. To find additional distal mappings, we also analyzed consensus distal alignments of loose end-associated reads. We then labeled loose ends as ‘fully mapped’ or ‘ambiguously mapped’, respectively, if they had a unique or ambiguous tumor-specific distal mapping and ‘partially mapped’ otherwise. See Supplementary Note 5 for full details of loose end classification.

### Neotelomere analysis and validation

We identified telomere repeat-positive sequences as those matching one of a panel of G-rich and C-rich telomere repeat trimers and their six cyclic permutations. A loose end was considered telomere repeat-positive if a tumor-specific telomere repeat-positive contig (see above) was found at the loose end. Given an sLRS telomere repeat-positive loose end, we counted read pairs comprising exclusively telomere repeats across all sLRS barcodes associated with the locus and multiplied the maximum value by the median intramolecular distance between reads across all molecules in that sLRS library to estimate the neotelomere length. To validate neotelomere candidates, genomic DNA was isolated from U2OS and Saos-2 cells and, where indicated, treated with Bal-31 exonuclease^24^. Bal-31-digested DNA was isolated by phenol extraction and ethanol precipitation and was then digested with the appropriate restriction enzyme. Gel electrophoresis of the DNA, Southern blotting and hybridization with Klenow-labeled radioactive probes were performed. See Supplementary Note 5 for additional details of neotelomere analysis and validation.

### Nominating NAHR junctions

We identified all pairs of sequences with ≥500 bp of homology (96% sequence identity) through exhaustive BWA-mem self-alignment of all 101-mers on both strands of GRCh37. Loose ends *b*_1_ and *b*_2_ were annotated as a putative NAHR junction if a sequence of ≥500 bp within 10 kb of *b*_1_ on its forward strand was found to be homologous to a sequence within 10 kb of *b*_2_ on its negative strand (Extended Data Fig. 10a). Similarly, junctions were annotated as NAHR if their breakends *b*_1_ and *b*_2_ demonstrated the above property.

### Distinguishing mechanisms of UPD

After allelic CN inference using JaBbA or other tools, UPD segments (total CN = 2 and minor allele CN = 0) were identified. UPD segments reference adjacent to another segment of CN = 2 without LOH (minor allele CN = 1) were called AHR-UPD; otherwise, segments were called P-UPD.

### Simulating tumor and normal SV profiles

We simulated 500 SRS whole-genome SV profiles on GRCh37 by rearranging the fully phased NA12878 Platinum genome^61^. To simulate phased rearrangement junctions, we randomly sampled and shifted pan-cancer SvAbA junctions^4^ and assigned each a random NA12878 haplotype. We also simulated NAHR junctions at a prevalence of 0.1–10% by linking pairs of homologous positions in the genome. Junction breakends were used to define allelic segments, and both were assigned a phased integer CN (‘balance’ function, gGnome^4^) with a target ploidy. We sampled junctions to simulate imperfect sensitivity for junction detection (accounting for sampling effects due to finite read depth or stromal admixture) at a rate proportional to tumor purity. Realistic purity and ploidy values were sampled from pan-cancer distributions^4^. To simulate read depth at bins or SNPs, the normalized purity-adjusted CN of each 1-kb bin was multiplied by a coverage factor to achieve a target genome-wide per-base read depth (80× in tumors and 40× in normal samples) and a bias factor was computed from normalized read counts for that bin in a random normal diploid sample. This product was used as the mean parameter for a Poisson distribution, which was sampled to obtain the final total (or allelic) read depth. See Supplementary Note 6 for additional simulation details.

### Benchmarking

To benchmark breakend detection, we compared the endpoints of simulated ‘ground-truth’ breakends to CN calls from JaBbA v1 (this paper), JaBbA v0.1 (ref. ^4^), ASCAT (v2.5.2)^14^, FACETS (v0.6.2)^17^, Sequenza (v3.0)^16^ and TITAN (v1.28)^15^. True-positive breakends were defined as those found within 10 kb of a ground-truth breakend on the same strand. We applied a similar approach to assess true-positive rates among JaBbA v1 versus v0.1 loose ends. To assess the accuracy of total CN inference, we computed the root mean square error between estimated and ground-truth total (or allelic) CN values across 10-kb genomic bins. See Supplementary Note 6 for additional benchmarking details.

### Identifying NAHR-eligible sites in T2T CHM13 v2

We sampled 1 million 500-bp substrings from T2T CHM13 and realigned them to T2T CHM13 using BWA-mem, identifying all alignments with cigar 500M. This yielded nearly 9 million position pairs (*p*_1_, *p*_2_), where *p*_1_ and *p*_2_ represent the starting coordinate of the query and alignment, respectively. We then divided the self-alignments into three categories (CNU × CNU, CNM × CNU and CNM × CNM) on the basis of the overlap of *p*_1_ and *p*_2_ with a CN-mappable region lifted from GRCh37 to T2T CHM13. See Supplementary Note 7 for details of T2T CHM13 NAHR analysis.

### Estimating the genome-wide unmappable breakend fraction

To extrapolate SRS findings from the CN-mappable to the CN-unmappable genome, we applied two principles. First, NAHR rearrangements occur in proportion to the number of homologous position pairs in the genome. Second, non-NAHR rearrangements (including AHR and non-HR SVs including end joining) occur in proportion to the number of bases in the genome. Our CN-mappable NAHR analysis found 216 somatic NAHR events across 2.8 × 10^8^ NAHR-eligible position pairs in 1,330 genomes, giving a somatic NAHR density of 6 × 10^−10^ events per bp^2^. Given the 2.7 × 10^10^ NAHR positions in the 13% of the genome that is CN-unmappable, we estimate an approximate NAHR burden of 16.2 breakends per tumor genome. Given the 681 AHR and 357,000 non-HR CN-mappable breakends in the 1,330 tumor samples, we estimate 0.6 AHR and 310 non-HR events per genome. Putting these numbers together, we estimate that ~17% of large SV breakends occur in CN-unmappable regions, and, given JaBbA’s 96% recall in CN-mappable regions, 80% of large SV breakends will be detected and 73% will be fully resolved by SRS. Given an estimated HR burden of 16.8 SVs per tumor genome, the fractional HR SV burden is approximately 5%. See Supplementary Note 7 for additional details of these calculations.

### Statistics and reproducibility

All statistical analysis was performed as stated in the figure legends using the R programming language (v4.0.2). Statistical methods were not used to predetermine sample size. The study design did not involve blinding or randomization. See Supplementary Note 8 for additional details on statistics and reproducibility as well as loose end association analyses.

### Reporting summary

Further information on research design is available in the Nature Portfolio Reporting Summary linked to this article.

## Online content

Any methods, additional references, Nature Portfolio reporting summaries, source data, extended data, supplementary information, acknowledgements, peer review information; details of author contributions and competing interests; and statements of data and code availability are available at 10.1038/s41588-023-01540-6.

### Supplementary information

Supplementary Information Supplementary Notes 1–8.

Reporting Summary

### Source data

Source Data Fig. 1 Statistical source data for Fig. 1.

Source Data Fig. 2 Statistical source data for Fig. 2.

Source Data Fig. 3 Statistical source data for Fig. 3.

Source Data Fig. 4 Statistical source data for Fig. 4.

Source Data Fig. 5 Statistical source data for Fig. 5.

Source Data Extended Data Fig. 1 Statistical source data for Extended Data Fig. 1.

Source Data Extended Data Fig. 3 Statistical source data for Extended Data Fig. 3.

Source Data Extended Data Fig. 4 Statistical source data for Extended Data Fig. 4.

Source Data Extended Data Fig. 5 Statistical source data for Extended Data Fig. 5.

Source Data Extended Data Fig. 6 Statistical source data for Extended Data Fig. 6.

Source Data Extended Data Fig. 8 Statistical source data for Extended Data Fig. 8.

Source Data Extended Data Fig. 9 Statistical source data for Extended Data Fig. 9.

Source Data Extended Data Fig. 10 Statistical source data for Extended Data Fig. 10.

## Data Availability

FASTA files for the GRCh37 and GRCh38 reference genomes were downloaded from the Genome Reference Consortium (GRCh37, https://www.ncbi.nlm.nih.gov/datasets/genome/GCF_000001405.13/; GRCh38, https://www.ncbi.nlm.nih.gov/assembly/GCF_000001405.40). The T2T CHM13 v2.0 reference was downloaded from the T2T Consortium (https://s3-us-west-2.amazonaws.com/human-pangenomics/T2T/CHM13/assemblies/analysis_set/chm13v2.0.fa.gz). Chain files for lifting hg19 and GRCh38 to T2T were downloaded from the T2T Consortium (GRCh37, https://s3-us-west-2.amazonaws.com/human-pangenomics/T2T/CHM13/assemblies/chain/v1_nflo/hg19-chm13v2.chain; GRCh38, https://s3-us-west-2.amazonaws.com/human-pangenomics/T2T/CHM13/assemblies/chain/v1_nflo/grch38-chm13v2.chain). SRS and LRS alignments for the LRS cohort have been deposited in the European Genome-phenome Archive (EGA), which is hosted by the European Bioinformatics Institute (EBI) and the Centre for Genomic Regulation (CRG), under accession number EGAD00001011047. SRS and sLRS alignments for the sLRS breast cancer cohort have been deposited at EGA under accession number EGAD00001010326. Data access requests will be centrally reviewed by a data access committee at NYU Langone Health and MSKCC. sLRS cell line data were deposited under NCBI Bioproject PRJNA623129. Whole-genome SRS alignments for cell lines used in the study were obtained from a previous study^55^ and are available through the Cancer Cell Line Encyclopedia (https://portals.broadinstitute.org/ccle). Pan-cancer analysis was performed on SRS whole-genome alignments previously curated and processed by Hadi et al.^4^. The majority of these data are available from The Cancer Genome Atlas (TCGA) Research Network consortium through the Database of Genotypes and Phenotypes (dbGaP; https://dbgap.ncbi.nlm.nih.gov/; accession ID phs000178.v11.p8) and the International Cancer Genome Consortium through the EGA (accession IDs EGAS00001001178, EGAS00001001552, EGAD00001004417 and EGAD00001002123). The remaining SRS whole-genome profiles used in this analysis are for lung adenocarcinomas available at EGA (EGAS00001002801)^62^, NYGC-IBM Cancer Alliance pan-cancer samples available at EGA (EGAS00001004013)^4^, Barrett’s esophagus samples available at dbGaP (phs001912.v1.p1)^63^ and prostate cancer samples available at dbGaP (phs000447.v1.p1)^64^. Figure source data are available with this manuscript and at https://github.com/mskilab/loose_ends_2023/tree/main/notebooks/source_data (GitHub), along with https://github.com/mskilab/loose_ends_2023/blob/main/notebooks/figures.ipynb (code for generating figure panels from the provided source data). We have supplied our 101-mer mappability track for GRCh37 online at https://github.com/mskilab/loose_ends_2023/blob/main/hg19.101.mappability.txt.gz. Source data are provided with this paper.

## Code availability

All statistical analyses and visualizations were performed using R (4.0.2) with Bioconductor (3.8; https://bioconductor.org/news/bioc_3_8_release/). Specific R/Bioconductor packages used included GenomicRanges for manipulation of genomic intervals, ComplexHeatmap for heatmap data, and dplyr and data.table for tabular operations. JaBbA uses the IBM CPLEX v12.6.2 optimizer, which is available under academic licensing (https://www.ibm.com/analytics/cplex-optimizer). All plots with the exception of track data and genome graph vertex illustrations were visualized using the ggplot and graphics R packages. Genomic tracks were plotted using the gTrack R package (https://github.com/mskilab-org/gTrack). Code and source data for generating the main figures and Extended Data figures are available in the following GitHub repository: https://github.com/mskilab/loose_ends_2023. An R package (loosends) for classifying JaBbA loose ends is available at https://github.com/mskilab-org/loosends. Genome graph analysis was performed using the packages JaBbA (https://github.com/mskilab-org/JaBbA) and gGnome (https://github.com/mskilab-org/gGnome). This included complex SV calling and haplotype (walk) reconstruction in balanced genome graphs, as described in Hadi et al.^4^. A Nextflow (https://www.nextflow.io/) module implementing the full JaBbA v1 pipeline is available at https://github.com/mskilab-org/nf-jabba.
