# Supplementary material for: Most large structural variants in cancer genomes can be detected without long reads
Source: Nat Genet. 2023 Nov 9;55(12):2139–48. doi: 10.1038/s41588-023-01540-6 (PMC10703688; doi:10.1038/s41588-023-01540-6)
Supplement: Supplementary file 1 — Supplementary Notes 1–8. [file 41588_2023_1540_MOESM1_ESM.pdf]

nature genetics

Article https://doi.org/10.1038/s41588-023-01540-6

Most large structural variants in cancer genomes can be detected without long reads

In the format provided by the authors and unedited

Supplementary Information

Supplementary Note 1.

Genome graph structure. A genome graph is a directed graph $G = (V, E)$ whose vertices $v_1, v_2 \in V$ represent strands of chromosomal segments and edges $e = (v_1, v_2) \in E$ represent segmental adjacencies. Each vertex $v \in V$ and edge $e \in E$ has a reverse complement vertex $\bar{v} \in V$ and edge $\bar{e} \in E$, respectively. Vertices $V = V_I \cup V_N$ comprise interstitial vertices V_I and *ends* V_N. The ends $V_N = V_T \cup V_L$ further comprise reference chromosome ends V_T and *loose ends* V_L. Edges $E = E_R \cup E_A \cup E_L$ comprise reference edges E_R, variant edges E_A, and loose end edges E_L. Loose end edges connect each interstitial vertex $v \in V_I$ to one incoming and one outgoing loose end in V_L. We use parentheses and superscripts to refer to the incoming and outgoing edges of specific vertices, e.g. $E_L^-(v) = E_L \cap E^-(v)$ and $E_L^+(v) = E_L \cap E^+(v)$ respectively denote the loose end edge that is upstream or downstream of a vertex v.

JaBbA statistical model. The JaBbA mathematical formulation used in this paper is an updated version of the algorithm presented in Hadi et al, 2020.[1] The JaBbA algorithm infers balanced genome graphs from junctions and breakends obtained through the analysis of cancer whole genome sequencing (WGS) by fitting integer vertex and edge weights to binned WGS read depth data through the solution of a MIP. To balance a genome graph, we define a mapping $\kappa : \{V \cup E\} \rightarrow \mathbb{N}$ of non-negative integer CN to vertices and edges of G, where $\kappa(v)$ and $\kappa(e)$ represent the CN of vertex $v \in V$ and edge $e \in E$, respectively. The principle of *junction balance* constrains the CN of every vertex to be equal to the sum of its incoming edges and the sum of its outgoing edges. Formally, the junction balance constraint is stated as follows:

$$\kappa(v) = \sum_{e \in E^-(v)} \kappa(e) = \sum_{e \in E^+(v)} \kappa(e) \tag{1}$$

In addition we require the CN κ to obey *skew-symmetry*, which means that every vertex and edge must have the same copy number as its reverse complement.

$$\kappa(v) = \kappa(\bar{v}),\ \forall_{v \in V} \quad \kappa(e) = \kappa(\bar{e}),\ \forall_{e \in E} \tag{2}$$

We call the combination (G, κ) for which κ satisfies Eqs. 1-2 a balanced genome graph. We infer balanced genome graphs from a genome graph G and binned, normalized, and purity / ploidy-transformed read depth data $x \in \mathbb{R}^n$ across n genomic bins (see below for read depth transformation details) through the solution of a mixed integer program (MIP), which assigns an integer CN $\kappa : V \cup E \rightarrow \mathbb{N}$ to the vertices and edges of G. The genome graph G is generated from a preliminary segmentation of genome-wide read depth (e.g. via CBS[2]) and a set of junctions from an SV caller (e.g. SvABA[3]). Additional details of genome graph construction are provided in Hadi et al 2020.[1]

Each vertex $v \in V_I(G)$ is associated with a partition of bins $J(v) \subseteq \{1, \ldots, n\}$ (based on genomic coordinate overlap) and a mean bin value $\rho(v, x, J) = \frac{1}{|J(v)|} \sum_{j \in J(v)} x_j$. We model each bin subset $x_{J(v)}$ as an i.i.d. sample from a Laplace distribution with scale parameter b and mean $\kappa(v)$. This is a change from our previous formulation, which modeled read depth per bin as samples from a Gaussian distribution. This reformulation allows us to linearize the objective function and express our optimization problem as a linear instead of quadratic MIP, improving convergence. The log likelihood is:

$$logP(x_{J(v)} | \kappa(v), b) = \sum_{j \in J(v)} log\ \mathcal{L}(x_j | \kappa(v), b) \tag{3}$$

where $\mathcal{L}(\mu, b)$ is the Laplace probability density function with mean μ and scale parameter b. The scale parameter b models read depth noise. As variance in read depth noise is expected to be proportional to the copy number, we set this parameter as $b(v, x, J) = max(1, \sqrt{\rho(v, x, J)})$ for each vertex v.

Given this model, the joint log-likelihood of the read depth data x across the graph given copy number assignment κ is

$$logP(x|\kappa) = Const(\kappa) - \sum_{v \in V_I} \frac{|J(v)|}{b} |\rho(v, x, J) - \kappa(v)| \tag{4}$$

We also refer to $\mathcal{V}(G, \kappa, x, J) = \sum_{v \in V_I} \frac{|J(v)|}{b} |\rho(v, x, J) - \kappa(v)|$ as the *read depth residual* of the balanced genome graph (G, κ) relative to data x.

Junction balance and skew-symmetry constraints in Eq. 1-2 may require nonzero copy number to be placed at one or more loose end edges. Each loose end in the input graph represents a slack variable that allows the junction balance constraint to be relaxed at specific internal vertices, allowing the data to be fit even when junctions are missing from the input (e.g. due to low mappability, sequencing depth, or purity). Only loose ends that are given nonzero CN are considered to be "present" in the final graph. To penalize solutions that require the use of many loose ends, we add an exponential prior with decay parameter λ on the loose end count (the number of loose ends with CN > 0) in (G, κ), which makes models with many missing junctions unlikely. This prior has log likelihood

$$logP(\kappa|G, \lambda) = -|V| log\lambda - \lambda\mathcal{R}(G, \kappa) \tag{5}$$

where

$$\mathcal{R}(G,\kappa) = \sum_{v \in V_I} \mathbb{1}_{\kappa(E_L^-(v))>0} + \mathbb{1}_{\kappa(E_L^+(v))>0} \quad \textbf{(6)}$$

is a *loose end penalty*. Adding the log likelihood in Eq. 4 to the prior in Eq. 5 yields a penalized log likelihood for the data with regularization parameter λ. Under this model, the maximum a posteriori probability estimate of κ will minimize the function

$$f(G,\kappa,x,J,\lambda) = \mathcal{V}(G,\kappa,x,J) + \lambda\mathcal{R}(G,\kappa) \quad \textbf{(7)}$$

which combines the read depth residual $\mathcal{V}$ and ℓ_0-norm loose end penalty $\mathcal{R}$ into a single piecewise-linear objective. We use f to define a linear MIP, which we solve to infer a maximum a posteriori estimate for κ given data x and genome graph G.

$$\begin{aligned} \underset{\kappa:V\cup E\to\mathbb{N}}{\text{minimize}} \quad & f(G,\kappa,x,J,\lambda) \\ \text{subject to} \quad & \kappa(v) = \kappa(\bar{v}),\ \forall_{v\in V} \\ & \kappa(e) = \kappa(\bar{e}),\ \forall_{e\in E} \\ & \kappa(v) = \sum_{e\in E^-(v)} \kappa(e) \quad = \sum_{e\in E^+(v)} \kappa(e), \forall_{v\in V} \\ & \kappa(e) > 0,\ \forall_{e\in E_F} \end{aligned} \quad \textbf{(8)}$$

where $E_F \subseteq E$ are a user-specified subset of edges to force incorporate into the graph (e.g. high confidence junctions). The solution of Eq.8 yields a balanced genome graph (G,κ) which maximizes the probability of read depth data while minimizing the number of utilized loose ends (i.e. with CN>0). The only hyperparameter in this inference is λ, which sets the prior probability for a loose end at a segment. After applying some hyperparameter tuning (data not shown), we have set λ to 20.

Allelic mass balance. Given a balanced genome graph (G,κ) representing total CN across the genome, we construct an associated balanced *allelic* genome graph $(\hat{G},\hat{\kappa})$ where $\hat{G} = (\hat{V},\hat{E})$. In $\hat{G}$, each vertex $v \in V$ from G gives rise to two allelic nodes $\hat{v}^h, \hat{v}^l \in \hat{V}$ representing major and minor parental alleles, respectively, of v. By convention, the CN of the major allele is greater than or equal to that of the minor allele. Similarly, each edge $e \in E$ from G gives rise to four edges $\hat{e}^{hh}$, $\hat{e}^{hl}$, $\hat{e}^{lh}$, $\hat{e}^{ll} \in \hat{E}$ joining each possible pair of adjacent major and minor alleles.

We track the mapping between nodes and edges of G and $\hat{G}$ with the function $p : \hat{V} \cup \hat{E} \to V \cup E$, e.g. $v = p(\hat{v}^h)$ and $e = p(\hat{e}^{hh})$ in the examples above. We also track the major and minor allele status of each vertex $\hat{v} \in \hat{V}$ via the function $q : \hat{V} \to \{0,1\}$ such that $q(\hat{v})$ is equal to 0 if $\hat{v}$ is a minor allele vertex and 1 if it is a major allele vertex.

The goal of allelic genome graph balancing is to find a mapping $\hat{\kappa} : \hat{V}_I \cup \hat{E} \to \mathbb{N}$ that is (1) consistent with the original (total CN) balanced genome graph (G,κ) while (2) satisfying the infinite sites model of molecular evolution and (3) maximizing the probability of heterozygous SNP allelic read depth. The first requirement constrains the CN $\hat{\kappa}(\hat{v})$ of all allelic vertices in $\hat{v} \in \hat{V}$ associated with a given interstitial vertex $v \in V_I$ to sum to the CN of that vertex, and analogously for variant edges. The second requirement states that every variant junction occurred at exactly a single time point in evolution, and hence on a single parental allele. This allows at most one of the four possible variant edges and at most one of the two reference edges upstream (or similarly downstream) of a vertex to have nonzero copy number. To address the infinite sites model we add constraints as well as a function $\phi : \hat{v} \in \hat{V} \to \{0,1\}$ to represent the parental chromosomal *phase* of each allelic vertex, which is inferred during optimization along with the CN $\hat{\kappa}$.

The third requirement is addressed by an objective function $\hat{f}$ that, similar to above, assesses the likelihood of the observed (allelic) read depth given an (allelic) CN assignment. As input, $\hat{f}$ takes a matrix $\hat{X} \in \mathbb{R}^{n\times 2}$ of purity / ploidy-transformed major and minor allelic read depth at m germline heterozygous SNPs (see Supplementary Note 2 for derivation). Given $\hat{J}(\hat{v}) \subseteq \{1,\ldots,m\}$, which maps vertices $\hat{v} \in \hat{V}_I$ to indices of overlapping SNPs, we can compute mean read depth $\rho(\hat{v},\hat{X},\hat{J}) = \frac{1}{|\hat{J}(\hat{v})|}\sum_{j\in\hat{J}(\hat{v})} \hat{X}_{\hat{J}(\hat{v}),q(\hat{v})}$. Analogous to the total CN analysis, we model the log likelihood as a Laplace probability density with mean $\hat{\kappa}(\hat{v})$ and scale parameter $b(\hat{v},\hat{X},\hat{J}) = max(1, \sqrt{\rho(\hat{v},\hat{X},\hat{J})})$, which yields a read depth residual $\hat{\mathcal{V}}(\hat{v},\hat{\kappa},\hat{X},\hat{J}) = \sum_{\hat{v}\in\hat{V}_I} \frac{|\hat{J}(\hat{v})|}{b(\hat{v},\hat{X},\hat{J})} |\rho(\hat{v},\hat{X},\hat{J}) - \kappa(v)|$ across all interstitial allelic vertices. We combine this with an ℓ_0-norm penalty on the number of allelic loose ends $\hat{\mathcal{R}}(\hat{G},\hat{\kappa}) = \sum_{\hat{v}\in\hat{V}_I} \mathbb{1}_{\kappa(\hat{E}_L^-(\hat{v}))>0} + \mathbb{1}_{\kappa(E_L^+(\hat{v}))>0}$, which gives a piecewise-linear objective function $\hat{f}(\hat{G},\hat{\kappa},\hat{X},\hat{J},\hat{\lambda}) = \hat{\mathcal{V}}(\hat{v},\hat{\kappa},\hat{X},\hat{J}) + \hat{\lambda}\hat{\mathcal{R}}(\hat{G},\hat{\kappa})$.

Putting this together, given G, κ, $\hat{G}$, $\hat{J}$, $\hat{X}$, p, and q, we obtain the maximum a posteriori estimate for allelic CN $\hat{\kappa}$ and phase

ϕ by solving the optimization:

$$
\begin{aligned}
\underset{\hat{\kappa}:\, \hat{V}_I \cup \hat{E} \to \mathbb{N},\ \phi:\, \hat{V}_I \to \{0,1\}}{\text{minimize}} \quad & \hat{f}(\hat{G}, \hat{\kappa}, \hat{X}, \hat{J}, \hat{\lambda}) \\
\text{subject to} \quad & \hat{\kappa}(\hat{v}) = \hat{\kappa}(\bar{\hat{v}}),\ \forall_{\hat{v} \in \hat{V}} \\
& \hat{\kappa}(\hat{e}) = \hat{\kappa}(\bar{\hat{e}}),\ \forall_{\hat{e} \in \hat{E}} \\
& \hat{\kappa}(\hat{v}) = \sum_{\hat{e} \in \hat{E}^-(\hat{v})} \hat{\kappa}(\hat{e}) = \sum_{\hat{e} \in E^+(\hat{v})} \hat{\kappa}(\hat{e}),\ \forall_{\hat{v} \in \hat{V}} \\
& \kappa(v) = \sum_{\hat{v} \in \hat{V} \mid p(\hat{v}) = v} \hat{\kappa}(\hat{v}),\ \forall_{v \in V_I} \\
& \kappa(e) = \sum_{\hat{e} \in \hat{E} \mid p(\hat{e}) = e} \hat{\kappa}(\hat{e}),\ \forall_{e \in E_A} \\
& \hat{\kappa}(\hat{v}_1) \geq \hat{\kappa}(\hat{v}_2),\ \forall_{\hat{v}_1, \hat{v}_2 \in \hat{V},\ v \in V \mid v = p(\hat{v}_1) = p(\hat{v}_2), q(\hat{v}_1) > q(\hat{v}_2)} \\
& \sum_{\hat{e} \in \hat{E} \mid p(\hat{e}) = e} \mathbb{1}_{\hat{\kappa}(\hat{e}) > 0} \leq 1,\ \forall_{e \in E_A} \\
& \sum_{\hat{e} \in \hat{E}_R^+(\hat{v})} \mathbb{1}_{\hat{\kappa}(\hat{e}) > 0} \leq 1,\ \forall_{\hat{v} \in \hat{V}_I} \\
& \sum_{\hat{e} \in \hat{E}_R^-(\hat{v})} \mathbb{1}_{\hat{\kappa}(\hat{e}) > 0} \leq 1,\ \forall_{\hat{v} \in \hat{V}_I} \\
& |\phi(\hat{v}_1) - \phi(\hat{v}_2)| < \mathbb{1}_{\hat{\kappa}(\hat{e}) > 0},\ \forall_{\hat{v}_1, \hat{v}_2, \hat{e} \mid \hat{e} = (\hat{v}_1, \hat{v}_2) \in \hat{E}_R}
\end{aligned}
\tag{9}
$$

where subscripts and superscripts on $\hat{E}$ are used similarly as for E above, e.g. $\hat{E}_A$ and $\hat{E}_R$ denote variant and reference edges, respectively, in the set $\hat{E}$ in $\hat{G}$. Similarly, $\hat{E}_R^-(\hat{v})$ and $\hat{E}_R^+(\hat{v})$ denote incoming and outgoing reference edges, respectively, to the allelic vertex $\hat{v}$. Since $\hat{f}$ is piecewise-linear, Eq. 9 can also be solved as a linear MIP. As in total CN genome graph balancing, the only hyperparameter in this optimization is $\hat{\lambda}$, which determines the prior probability for a loose end at a segment. After applying some hyperparameter tuning (data not shown), we have set $\hat{\lambda}$ to 20.

Supplementary Note 2.

Dryclean algorithm. We applied the dryclean algorithm[4] to 1 kbp binned read depth obtained via fragCounter (http://github.com/mskilab/fragCounter) to mitigate the effects of read depth fluctuation due to dosage-independent factors, including replication timing and GC-content. As input to dryclean we used a panel of normals (PON), comprising 1 kbp binned read depth profiles from normal diploid samples in our cohort.

The intuition behind dryclean is that non-dosage related effects on read depth are likely to be shared across many samples, while read depth changes due to dosage (CNVs) are likely to be private to a few samples. Applying this intuition, dryclean uses robust principal components analysis (rPCA) to project the read depth profile of a sample of interest (e.g. a tumor sample) on a low rank subspace inferred from variation in read depth data across a large panel (380 samples) of normal diploid samples (PON). To generate this low rank subspace, dryclean models a log read depth matrix of n samples across m genomic bins $M \in \mathbb{R}^{m \times n}$ as the sum of the low rank matrix $B \in \mathbb{R}^{m \times n}$ and a sparse matrix $F \in \mathbb{R}^{m \times n}$, by solving the following optimization problem:

$$\texttt{minimize}_{B,F} ||B||_* + ||F||_1 \tag{10}$$

subject to

$$M = B + F \tag{11}$$

where for a matrix A, $||A||_* = \sum_i \sigma_i$ refers to the nuclear norm of A (the convex relaxation of rank) and $||A||_1 = \sum_{ij} |a_{ij}|$ is the ℓ_1-norm of A. For each tumor sample, the projection of the log read depth profile onto B is then subtracted, and the remaining foreground signal is used as the input to JaBbA after exponentiation. We show an example of the read depth profile before and after dryclean correction in **Extended Data Fig. 1a**.

Purity and ploidy transformation. We obtained ploidy estimates for tumors with a matched normal WGS profile using ASCAT[5,6] followed by purity estimation were obtained via grid search (using the ppgrid function in the JaBbA package, http://github.com/mskilab/JaBbA).[1] For samples without a matched normal (e.g. cancer cell lines), purity and ploidy were

both obtained using ppgrid on binned total read depth, since, unlike ASCAT, ppgrid does not require heterozygous SNPs. We then used these purity and ploidy estimates to transform total and/or allelic read depth into units of clonal CN per cell.

Given a read depth vector $y_j,\ j \in 1,\ldots,n$ normalized such that $\frac{1}{n}\sum_{j=1}^{n} y_j = 1$, we apply the formula

$$x_j = \frac{2y_j - c_j\gamma}{2\beta} \tag{12}$$

where

$$\beta = \frac{\alpha}{\alpha\tau + 2(1-\alpha)} \tag{13}$$

and

$$\gamma = \frac{2(1-\alpha)}{\alpha\tau + 2(1-\alpha)} \tag{14}$$

where α denotes purity, τ denotes ploidy, and c_j denotes constitutional CN at bin j. Of note, c_j is two for autosomes and either one or two for sex chromosomes (depending on sex). The result x_j is in units of clonal CN per cell.

To transform major and minor allelic counts, we take a similar approach. We begin with a matrix of read counts $\hat{Y} \in \mathbb{R}^{m\times 2}$ at minor and major alleles respectively (i.e. where $\hat{Y}_{j,1} \leq \hat{Y}_{j,2}$) normalized such that $\frac{1}{2m}\sum_{j=1}^{m} \hat{Y}_{j,1} + \hat{Y}_{j,2} = 1$. To obtain $\hat{X} \in \mathbb{R}^{m\times 2}$ in units of clonal allelic CN we apply Eq. 12 with some modifications. To transform major allele counts we replace x_j with $\hat{X}_{j,2}$ and y_j with $\hat{Y}_{j,2}$ and set c_j to 1. For the minor allele, we apply the same calculation to obtain $\hat{X}_{j,1}$ from $\hat{Y}_{j,1}$ except we set c_j to 1 for autosomes and X chromosomes in females, and to 0 for X and Y chromosomes in males. Finally, a slightly different value of ploidy $\hat{\tau}$ is swapped in for τ in Eq. 14 in the calculation of γ. This value $\hat{\tau}$ is computed by averaging segmental CN values from the purity / ploidy fit across the m heterozygous SNP sites. A full derivation of this purity / ploidy transformation is provided in the Methods of Hadi et al., 2020.[1]

Chromosomal bias correction. In practice, the JaBbA pipeline employs three iterations of total CN MIP optimization (see Methods, JaBbA pipeline). The goal of the second iteration is to improve sensitivity, by incorporating lower confidence candidate junctions near loose ends. The goal of the final iteration is to improve specificity by correcting for non-integer read depth. In this final iteration, the node log-likelhood $\mathcal{L}$ in Eq. 3 is modified to include a chromosome-specific offset. Namely, given the reference chromosome of a vertex $C(v) \in 1\ldots,c$ (where $c = 25$ for the "standard" human chromosomes, namely 22 autosomes, X, Y, and M) we allow a chromosome specific offset $-0.5 \leq o(C(v)) \leq 0.5$ in the mean $\mu = \kappa(v) + o(C(v))$ of the Laplace distribution $\mathcal{L}(\mu, b)$ modeling vertex v. This modifies the read depth residual $\mathcal{V}(v,\kappa,x,J) = \sum_v \frac{|J(v)|}{b}|\rho(v,x,J) - \kappa(v) - o(C(v))|$ and objective function (Eq. 7). It also adds an additional set of variables and constraints (one per reference chromosome) $-0.5 \leq o(c) \leq 0.5, c \in \bigcup_{v\in V} C(v)$ to the MIP (Eq. 8). In practice we find that the addition of this offset to the MIP removes many loose ends that arise from small misestimates of purity / ploidy and subclonal CN alterations, two common causes of non-integer read depth.

AHR detection in allelic genome graphs. AHR involves a change in allelic CN without a change in total CN, hence AHR change-points can be hidden inside genome graph segments after total CN balancing. To enhance sensitivity for AHR we apply additional intra-segment change point detection by applying CBS[2] to vectors of minor allele counts within large ($\geq$ 1 Mbp) genomic segments belonging to the balanced genome graph produced after the final total CN MIP iteration in the JaBbA v1 pipeline. Any intra-segment changepoints detected by CBS are used to split the corresponding vertex in the total CN balanced genome graph. Specifically, each change point replaces that vertex v_1 with two new vertices v_2 and v_3 joined by a new reference edge (v_2, v_3), all which inherit the CN of v_1. The new vertices v_2 and v_3 also inherit, respectively, the incoming and outgoing edges of v_1. We do the same for the reverse complement vertex $\bar{v}_1$. It is trivial to show that these operations maintain mass balance, skew symmetry, and the value of f.

The resulting balanced genome graph is then used as input to allelic mass balance (Supplementary Note 1). Sites of AHR, which involve two reference adjacent segments v_2 and v_3 whose major and minor alleles both change their CN, will be fit by allelic mass balance as two reciprocal loose ends with CN>0, one downstream of v_2 and the other upstream of v_3. Though this approach identifies AHR with precision, we found that it was not sufficiently sensitive in practice, mainly because it adds a loose end penalty of 2λ to the allelic mass balance objective $\hat{f}$ which discourages AHR calls at segments that are smaller or reside in areas of lower SNP density. To increase sensitivity for AHR events, we take a slightly modified approach in practice, namely discounting the loose end penalty at intrasegment changepoint-associated loose ends to $\frac{\lambda}{2}$. This is equivalent to having a higher prior probability for loose ends at these AHR candidate loci. As part of allelic graph post-processing, we also replace reciprocal loose end pairs fit with CN>0 with a variant "crossover edge", which facilitates the annotation of AHR events in these graphs.

Supplementary Note 3.

SRS library preparation. SRS library preparation was performed using the Illumina TruSeq DNA PCR-free Library Preparation Kit in accordance with the manufacturer's instructions. Briefly, 1 microgram of DNA was sheared using a Covaris LE220 sonicator (adaptive focused acoustics). DNA fragments undergo end-repair, bead-based size selection, adenylation, and Illumina sequencing adapter ligation. For samples with less material, we used the Illumina TruSeq DNA Nano Library Preparation Kit and 100ng input, as described by the manufacturer's instructions. SRS was performed on an Illumina NovaSeq 6000 sequencer using 2x150bp cycles.

SRS data processing. SRS FASTQ files were aligned to the GrCh37 reference using BWA mem (v0.7.17). SvABA (https://github.com/walaj/svaba/releases/tag/1.1.0, v1.1.0) was used to call junctions. We computed read depth across 1 kbp bins using fragCounter (https://wwwgithub.com/mskilab/fragCounter). We assessed tumor and normal constitutional SNP counts at HapMap 3.3 sites (https://www.sanger.ac.uk/resources/downloads/human/hapmap3.html) using Rsamtools (R / Bioconductor) as any site with > 0.2 and < 0.8 ALT variant allele fraction in the matched normal sample. We applied JaBbA v0.1[1] and v1 (see below for details, https://github.com/mskilab/JaBbA) with inputs from fragCounter, SvAbA, and heterozygous SNP counts to infer junction balanced genome graphs (see below for details). We annotated graphs for SV events using gGnome (v1, https://github.com/mskilab/gGnome).[1] In addition to JaBbA v1 and v0.1[1] we ran additional SCNA callers (ASCAT,[5] FACETS,[7] sequenza,[8] TITAN[9]). To identify reciprocal SVs, we looked for clusters of junctions spanning at least 10 kbp with reciprocal breakends, which we defined as being within 1 kbp of another breakend in the opposite orientation. In addition to SVs, we called somatic and germline SNVs and indels using Strelka2 (v2.92)[10] under paired (i.e. tumor / normal) mode with default parameters. In addition to the recommended filters (FILTER=PASS), a universal mask (https://github.com/lh3/sgdp-fermi/releases/download/v1/sgdp-263-hs37d5.tgz) was used to remove common artifacts in low-mappability regions, described in Mallick et al.[11] Variant annotations were obtained for SNV/indel using SnpEff v4.3[12] (https://pcingola.github.io/SnpEff) with the GRCh37.75 reference database.

Reference Sequence and Annotation Sources. Cytoband and repeat tracks were obtained from the UCSC Genome Browser database. The repeat sequence and poly-A databases from TraFiC[13] (github download, `https://gitlab.com/mobilegenomesgroup/TraFiC/~/tree/multispecies/databases/hg19`) were supplemented with ribosomal reference sequences and satellite reference sequences from RefSeq (alpha-satellite consensus sequence X07685.1, gamma X satellite sequence X87951.1, ribosomal complete sequence U13369.1, beta-satellite sequence M25749.1) to comprise the repeat reference against which contigs were aligned. 6251 viral sequences were also obtained from RefSeq v1.1 (`ftp://ftp.ncbi.nlm.nih.gov/refseq/release/viral/`).

The T2T-CHM13v2.0 assembly with chrY supplemented from HG002 from Genome In A Bottle was downloaded using this link: `https://s3-us-west-2.amazonaws.com/human-pangenomics/T2T/CHM13/assemblies/analysis_set/chm13v2.0.fa.gz`. In addition, the chain file lifting the hg19 reference to the T2T assembly was downloaded using this link: `https://s3-us-west-2.amazonaws.com/human-pangenomics/T2T/CHM13/assemblies/chain/v1_nflo/hg19-chm13v2.chain`.[14]

Mappability analysis. We analyzed local patterns of base-level mappability to define base mappable and CN mappable regions. To assess base mappability, we performed self-alignment of a 101-mer starting at every position in the GrCh37 reference using the command `bwa mem -a -M`. We designated a position as base-unmappable if it had any full-length (`CIGAR 101M`) supplementary alignments, or if it contained any masked (`N`) bases. A position was designated *CN-unmappable* if more than 90% of the bases in a 1 kbp window around that position were base-unmappable; otherwise, the position was designated *CN-mappable*. As a result, CN-mappable positions include base-unmappable positions that did not harbor enough repetitive bases in their vicinity to be CN-unmappable. We have supplied our 101-mer mappability track for GrCh37 online at the following URL:(`https://github.com/mskilab/loose_ends_2023/blob/main/hg19.101.mappability.txt.gz`). We used a similar procedure to determine GrCh37 mappability at the scale of 150-mers, and to determine GrCh38 mappability.

Supplementary Note 4.

LRS library preparation. Long read sequencing was performed on the Oxford Nanopore Technologies (ONT) PromethION sequencer using R10 chemistry with two flow cells per tumor and one flow cell per normal sample. To obtain high molecular weight DNA for long-read DNA sequencing, DNA was extracted from tissue or blood using the MagAttract HMW DNA Kit (Qiagen). The kit enables purification of high molecular weight (100-200kb) DNA using an efficient combination of high-performance magnetic beads and silica-based chemistry. An RNase-step is included. DNA concentration (Qubit) and size range was assessed on the TapeStation (Agilent) or BioAnalyzer (Agilent) to determine if the amount and size of DNA is sufficient for long read sequencing.

LRS data processing. LRS SV junction calls were identified taking the two-way consensus of four callers: cuteSV (release v.2.0.2[15]), SAVANA (release 0.2.3[16]), SVIM (release 2.0.0),[17] and Sniffles2 (release v2.0.7[18]). These algorithms were run on

tumor and normal sample separately (CuteSV, SVIM, Sniffles2) or in paired mode (SAVANA). Junctions were merged across algorithms and matched tumor and normal pairs if both breakends were in the same orientation and overlapped within 1 kbp. Somatic junctions were then identified as any junction found by two or more algorithms in the tumor sample and not found in the matched normal. To increase sensitivity for reciprocal SV detection, we used all tumor-specific junction calls from all SV callers so the junctions involved in a reciprocal rearrangement need not be made by exactly the same caller.

sLRS library preparation. Additional sLRS whole genome profiles were obtained from a collection of ATCC (https://www.atcc.org/) cell lines by applying the above protocol (U2OS, NCI-H209, NCI-H661, A549), taking data from a previous study (NCI-H526, NCI-H838),[1] or downloading publicly available data from 10X Genomics (HCC1954, HCC1954BL, HCC1143, HCC1143BL, https://www.10xgenomics.com/resources/datasets). We note that all of these cell lines were also SRS profiled by the Cancer Cell Line Encyclopedia[19] (https://sites.broadinstitute.org/ccle/). For sLRS library preparation, high molecular weight (HMW) genomic DNA (gDNA) was extracted using a Qiagen MagAttract HMW DNA Kit (Qiagen, Germany) according to the manufacturers protocol. The HMW gDNA had an approximate mode length of 50 kbp and max length of 200 kbp, as estimated on a separate 75V pulse-field gel electrophoresis (BluePippin 5-430kbp protocol). HMW DNA was then subjected to sLRS library preparation using a 10X Genomics Chromium Genome Library Kit v2 (Lot 152527, 10X Genomics) following the Chromium Genome Reagent Kits v2 User Guide. sLRS libraries were sequenced on an Illumina NovaSeq 6000 Sequencing System (Illumina, San Diego, CA) with S4 flow cells to approximately 30X average read depth and approximately 170X physical coverage.

sLRS data processing. Similar to previous sLRS studies[20,21] we grouped linked reads sharing a barcode within 10 kbp of reach other into "read clouds". To nominate SV junctions, we applied a consensus of three algorithms (LinkedSV (https://github.com/WGLab/LinkedSV, commit 1b77a14),[22] GROC-SV (https://github.com/grocsvs/grocsvs, version 0.2.6),[20] and NAIBR (https://github.com/raphel-group/NAIBR, commit 15eba96)[21]) run on tumor and normal sLRS alignments. Briefly, tumor and normal junction calls across all algorithms were merged if their breakends were in the same orientation and overlapped within 10 kbp. Somatic SVs were then called as junctions found in tumors by two or more algorithms and not detected in the normal. We additionally called somatic and germline SNV and indels using `Strelka2`[10] under paired (i.e. tumor / normal) mode with default parameters. Germline haplotypes were obtained from Strelka2 germline SNV calls processed using HapCut2 (github.com/vibansal/HapCUT2). We used somatic and germline SNV, indel, SV, and haplotype calls to assess sLRS and SRS homologous recombination proficiency in the sLRS cohort on the basis of *BRCA1* and *BRCA2* mutation status. We found 10 homologous recombination proficient cancer samples (U2OS, NCI-H526, NCI-H838, NCI-661, NCI-HCC1954, HCC1143, four MSK samples with either mono-allelic or non-pathogenic mutations in *BRCA1* or *BRCA2*) and 21 homologous recombination deficient cancer samples (twelve tumors with biallelic pathogenic variants in BRCA1, and eight tumors with biallelic BRCA2 pathogenic variants, and one tumor with homozygous loss of BRCA2 in the MSKCC cohort). SRS and sLRS alignments for these samples have been been deposited at the European Genome-phenome Archive (EGA), which is hosted by the European Bioinformatics Institute (EBI) and the Centre for Genomic Regulation (CRG), under accession number EGAD00001010326. In summary, we analyzed 27 sLRS tumor normal pairs (25 cases from MSKCC, 2 cancer cell lines – HCC1143, HCC1954 - and their matched normal samples).

Supplementary Note 5.

Loose end mapping. To identify candidate distal mappings for loose ends, we used local assembly and realignment of loose end-associated reads, namely tumor and normal reads aligning to the positive strand within in the 5 kbp vicinity of each loose end, as well as their reverse-complemented mates. We applied local assembly to these reads across 20 overlapping bins (1 kbp width, 500 bp stride) around each loose end using Fermi[23] (https://github.com/lh3/fermi, v1.1) via the R package RSeqLib (https://github.com/mskilab-org/RSeqLib). The resulting contigs were assessed for tumor and normal read support through BWA re-alignment of reads to contigs and the reference (via the readsupport package, https://github.com/mskilab/readsupport) and identifying read pairs with superior alignment scores and/or shorter insert sizes to the contig. Tumor-specific contigs (i.e. those with twenty fold read support in tumor vs. normal, greater than three tumor supporting reads, and fewer than two normal supporting reads) were then aligned to a modified GRCh37 reference augmented with a repetitive element database containing containing consensus sequences for LINE-1, Alu, SVA, ERVK, polyA, ribosomal, and satellite elements, and 6251 viral genome sequence. Contigs with distal alignments (i.e. those aligning further than 1 kbp from the loose end positive strand, including the negative strand of the same loose end) were then used for loose end classification (see below). To find additional mappings, we also analyzed consensus distal alignments of loose end-associated reads. Namely, we identified all distal regions (i.e. those further than 10 kbp from the loose end positive strand) harboring ≥ 3 tumor, ≤ 2 normal, and ≥ 20 fold more tumor than normal alignments among loose-end associated reads. We then annotated each region with its maximal mapping quality across all loose-end associated reads.

Loose end classification. We classified loose ends as somatic vs. germline, and further classified somatic loose ends as fully, ambiguously, or partially mapped on the basis of whether a unique, non-unique, or absent distal mapping, could be respectively

identified. We distinguished somatic from germline loose ends by analyzing matched normal read depth in the vicinity of the loose end. Specifically, we compared bins of matched normal CN-mappable read depth within 100 kbp upstream (negative coordinates) and downstream (positive coordinates) of each loose end. If there were were greater than five CN-mappable bins on each side and greater than >0.6 CN difference, we called the loose end "germline", otherwise we called it "somatic". We then further classified somatic loose ends on the basis of tumor and normal reads aligning within 5 kbp of the loose end on the forward strand. Each somatic loose ends were further classified as a partially, ambiguously, or fully mapped breakend on the basis of the distal mapping (see above). If the loose end was associated with a high mapping quality distal contig (MAPQ $\geq$ 50) or consensus alignment (MAPQ $>$ 50), the loose end was called "fully mapped". Otherwise, if the loose end was associated with any other distal contig or consensus alignment, it was called "ambiguously mapped". The remainder of loose ends were called "partially mapped".

Neotelomere analysis. We assessed loose end-associated reads and contigs for G-rich telomeric repeats (TTAGGG, TCAGGG, TGAGGG, and TTGGGG) and their C-rich reverse complements. We used the BioStrings package in R/Bioconductor to identify all loose end-associated telomere repeat-positive reads and contigs, as those matching one of 768 G-rich and C-rich repeat trimers and their 6 cyclic permutations. A loose end was considered telomeric repeat-positive if a tumor-specific telomere-repeat-positive contig was found with local assembly (see above) or if there were $\geq$ 3 tumor telomere repeat-positive reads, $\leq$ 2 normal telomere repeat-positive reads, and $\geq$ 20 ratio of tumor to normal telomere repeat-positive reads associated with the loose end. To assess telomere length in sLRS data, we defined telomere repeat-positive read pairs where both mates comprised exclusively telomere repeats and one read comprised G-rich and its mate comprised C-rich telomere repeats. We then multiplied the median genome-wide spacing between consecutive read pairs in linked read clouds (see above) by the number of fully telomeric reads per barcode to estimate telomere length.

Neotelomere validation. To validate neotelomere candidates on chromosome 10 and 12 in U2OS, genomic DNA was isolated from U2OS and SaOS cells and, where indicated, treated with Bal-31. Bal-31 digested DNA was isolated by phenol extraction and ethanol precipitation and then digested with the appropriate restriction enzyme. Gel-electrophoresis of the DNAs, Southern blotting (with acid treatment to promote transfer of large fragments), and hybridization with Klenow-labeled radio-active locus-specific probes was performed. Locus-specific probes were amplified from a cell line (HCC1143, ATCC) using primers L10F (TGGATGCCTCCTTACAAACTGG) and L10R (CAAGCAAATGTCGGTCCCAC) for the chromosome 10 neotelomere and primers L12F (ACTAAAGCCCGAGGAAAGGAG) and L12R (GAGTCTGTCATCCCAAGTAGTGG) for the chromosome 12 neotelomere.

Supplementary Note 6.

Simulating tumor and normal SV profiles. We simulated realistic fully phased total binned read depth, allelic counts, and SV junction profiles on GrCh37 by rearranging the fully phased NA12878 Platinum genome.[24] To simulate phased rearrangement junctions, we randomly sampled somatic junction clusters aggregated across a pan-cancer dataset[1] of SvAbA junctions. SVs were clustered using the `eclusters` method in the package `gGnome`. To modify junction coordinates from those in existing samples, we added a random shift to the endpoints of each junction cluster which was chosen uniformly from the range -10 Mbp to 10 Mbp (while staying within the boundaries of chromosomes). The number of selected junction clusters for each tumor sample was randomly sampled from the empirical distribution of junction clusters of each size k per sample, where most junction clusters comprised a single junction. Each junction cluster was then randomly assigned a parental haplotype (with each haplotype assigned with probably 0.5). To simulate NAHR junctions, we also randomly augmented the number of junctions by 0.1-10% by adding junctions with endpoints connecting homologous base pairs in the genome (> 500 bp and > 96% homology). Finally, junctions and genomic segments were assigned a phased integer copy number using the function `balance` in `gGnome` to a target ploidy sampled from a distribution of ploidies obtained from pan-cancer WGS analysis.[1]

To generate junction calls with imperfect sensitivity (either due to read depth or stromal admixture) we used random drop out according to a distribution obtained from a read level tumor genome simulation previously used for benchmarking in Hadi et al., 2020.[1] To estimate an appropriate drop out rate as a function of purity, we analyzed SvAbA calls from high coverage impure tumor and normal BAM files from the cancer cell line HCC1954 and its matched normal HCC1954BL. Simulated tumor BAM files were generating by admixing and subsampling tumor and normal reads according to a purity ladder (0.1 to 0.9, increments of 0.1) to a target 80X tumor genome read depth. We then assessed junction recall relative to the full coverage (120X) and purity = 1 data as a function of purity, and used this to compute an empirical distribution of junction drop-out as a function of purity to use in our simulation. In addition, we removed all junctions with either endpoint overlapping a base unmappable region. In general, this resulted in 5-60% of the true junctions missing from the simulated junction call set.

To simulate total binned read depth and allelic SNP counts, each sample was assigned a realistic purity between 0.1 and 1, was sampled from an empirical distribution of tumor purity values previously computed across our pan-cancer cohort.[1] The effective dosage of each genomic segment was then calculated using a purity-weighted average of tumor segment copy number and copy number (two for autosomes and either zero, one, two for sex chromosomes, based on randomly assigned sex).

To simulate reads per bin for a 1 Kbp genomic bin, the effective dosage of that bin was multiplied by the target sequencing coverage (80X for tumor samples and 40X for matched normal samples) and the sum of the bin width (1 Kbp) and the average read length (150 bp), and divided by the average read length. This number was in turn multiplied by a bias factor taken as the read counts for that bin in a random normal diploid sample. The goal of the bias factor was to model dosage-independent read depth "waviness" (due to replication timing, GC content, and other platform factors[4]). The read depth per bin was then sampled from a Poisson distribution parameterized by this mean. Heterozygous SNP counts were simulated in a similar way; however, the effective dosage for each SNP was calculated by using the allele-specific copy number of the high and low alleles. The sites of these reference SNPs were those available from HapMap.[25] A schematic summarizing the full simulation approach is shown in **Extended Data Fig. 3a**.

AHR simulation. We initially used 500 simulated tumor samples to benchmark the overall SV breakend calling efficacy; however, none of these genomes had AHR. We simulated an additional 400 samples to benchmark the accuracy of AHR calling. To model AHR, we introduced additional breakends between randomly selected gaps between junctions, and constrained loose ends on opposite parental homologs to have copy number one, and constrained total copy number to be two. We note that we did not have to explicitly model progressive uniparental disomy (P-UPD, **Fig. 4a**, **Extended Data Fig. 9**) or other LOH as these arose spontaneously during tumor genome simulation (see above). These comprised two hundred "matched" genomes with the same junctions, but where one genome contained an instance of AHR and the other did not. This was to check that biases in purity and junction burden did not drive efficacy of AHR calls.

Structural variant breakend detection accuracy. We benchmarked structural variant detection by comparing the endpoints of CNVs (JaBbA v0.1,[1] ASCAT,[5] FACETS,[7] sequenza,[8] TITAN[9]) and the location of loose ends/junctions in JaBbA to the breakends known to exist in the ground truth graph. We defined a true positive call as a call overlapping a ground truth call within 10 kbp and on the correct strand.

Copy number estimation accuracy. To assess the accuracy of total copy number inference, we divided the genome into 10 kbp bins and computed the average total copy number of the ground truth simulated data within each bin, as well as the average total copy number inferred by each CNV inference algorithm. We then computed the root mean square error across all genomic bins. Similarly, for allele-specific copy number inference, we compared the mean high and low allelic copy numbers per 10 kbp genomic bin with the ground truth, and computed the root mean square error (**Extended Data Fig. 3g**). For all methods (including ASCAT, sequenza, TITAN, and FACETS), we excluded bins overlapping CN unmappable regions by more than 90% of the 10 kbp bin width.

Loose end overlap with missing breakends. We compared the loose end calls made by JaBbA v1 and JaBbA v0.1 with the sites of breakends missing from the input to JaBbA (whether randomly dropped out due to purity or due to mappability). We denoted a true positive loose end call as one overlapping a missing breakend call within 10 kbp on the correct strand. We separated samples based on purity inferred by as part of the JaBbA pipeline (**Extended Data Fig. 3e-g**).

Supplementary Note 7.

Identifying NAHR eligible sites in T2T CHM13v2. We sampled one million 500 base pair substrings uniformly without replacement from the T2T CHM13 reference and realigned them to this reference using BWA mem, identifying all alignments with cigar `500M`. This yielded nearly 9 million position pairs (p_1, p_2) where p_1 is the starting base pair of the query sequence, and p_2 is the starting base pair of the alignment. To categorize pairs as belonging to CN-mappable versus CN-unmappable regions, we used the GrCh37 chain file provided by the T2T consortium to lift the CN mappable ranges from GrCh37 to T2T. We then divided the self-alignments into three categories (CNUxCNU, CNMxCNU, CNMxCNM) based on the overlap of p_1 and p_2 with a lifted CN-mappable region. Specifically, if both p_1 and p_2 were located in CN-mappable regions, then the pair was considered CNMxCNM; if both were in CN-unmappable regions, then the pair was considered CNUxCNU; and if one site was in a CN-mappable region and the other site was in a CN-unmappable region then the pair was considered to be CNMxCNU. We found 9.1×10^4 CNMxCNM pairs, 8.8×10^6 CNUxCNU pairs, and 5.0×10^4 CNMxCNU pairs. Since we sampled 1 million out of 3.05 billion possible positions, there should be approximately 2.8×10^8 CNMxCNM pairs, 2.7×10^{10} CNUxCNU pairs, and 1.5×10^8 CNMxCNU pairs. Overall, we estimate that there are approximately one hundred-fold more eligible NAHR pairs containing at least one breakend within a CN-unmappable region.

To visualize the distribution of these eligible sites in **Fig. 5b**, pairwise alignments were grouped into either 10 Mbp (left, genome-wide heatmap) or 1 Mbp (right, CN-unmappable heatmap) genomic bins, and the number of pairs falling in each bin were counted. The matrix of bin pairs was made symmetric by summation with its transpose to produce the displayed heatmaps.

Estimating genome-wide unmappable breakend fraction. To extrapolate SRS findings to the whole genome we applied two principles: First, rearrangements driven by NAHR occur in proportion to the number of homologous position pairs in the genome, i.e. those which are eligible for NAHR. Our calculations (see above, **Fig. 5b-c**) show that there are roughly 100-fold

more NAHR-eligible position pairs in CN-unmappable regions than in CN-mappable regions. As a result, we estimate there will be a 100-fold higher rate of NAHR in CN-unmappable regions relative to CN-mappable regions.

Second, rearrangements not driven by NAHR (such as non-homologous end joining) occur in proportion to the number of bases in the genome, regardless of whether those bases are CN mappable or unmappable. Notably, we treat AHR as a non-HR process, since it involves recombination between parental homologues, and thus there will be a similar number of opportunities for AHR in CN mappable and CN unmappable regions. As a result, AHR breakends should occur at similar densities (per bp) in CN mappable and CN unmappable regions.

Given these assumptions, let C represent the event that a breakend occurs in a CN-mappable region (as opposed to a CN-unmappable region). The first quantity we sought to estimate was $P(C)$, the fraction of breakends occurring in CN-mappable regions. In our cohort of 1330 samples, we observed 216 somatic NAHR breakends, 681 AHR breakends, and 357K non-HR (non-NAHR, non-AHR) breakends from junctions and loose ends not consistent with any homologous recombination event. Our analysis of the T2T reference indicates that there are approximately 2.8×10^8 eligible position pairs for NAHR within CN-mappable regions of the genome, giving a somatic NAHR density of $(216/1330)/(2.8 \times 10^8) = 6 \times 10^{-10}$ events per base pair^2 per tumor genome. Given one hundred times more eligible NAHR positions in the part of the genome that is CN-unmappable, we estimate an approximate NAHR burden of 16.2 breakends per genome.

For AHR breakends, we observed 681 such breakends across our cohort. If these occur at a similar density in CN-mappable versus CN-unmappable regions, then expected genome-wide burden of AHR breakends is $(681/1330)/0.87 = 0.6$. Finally, a similar calculation leads to an estimate of $(3.57 * 10^5)/1330/0.87 \approx 310$ non-NAHR breakends per genome. Putting these numbers together, we estimate that $\frac{0.13*(0.6+310)+16}{(0.6+310)+16.2} \approx 17\%$ of somatic breakends will occur within CN-unmappable regions, and $P(C) \approx 0.83$. Next, let J represent the event that a breakend can be detected by JaBbA, either as a loose end or junction. We would like to estimate the quantity $P(J) = P(J|C)P(C) + P(J|\bar{C})P(\bar{C})$. Notably $P(J|\bar{C})$ is zero, because CN-unmappable regions are masked to JaBbA. Within CN-mappable regions, the recall of JaBbA for somatic SV's is approximately 96%, so $P(J) \approx 0.96 * 0.83 \approx 0.80$. Finally, let M represent the event that an SRS breakend is fully mappable. We estimate the fraction of fully mapped breakends as $P(M) = P(M|J \cap C)P(J \cap C)$ since only breakends detected by JaBbA in CN-mappable regions can be fully mapped. Empirically, we find that this fraction is $\approx 90\%$, which leads us to our final estimate that $\approx 73\%$ of large SV's can be fully mapped by short reads. Given 310 non-HR, 16.2 NAHR, and 0.6 AHR SVs in the average cancer genome, our final estimate of the genome-wide proportion of HR-driven SVs to total SVs is $\frac{16.2}{16.2+310+0.6}$ or approximately 5%.

Supplementary Note 8.

Statistics and reproducibility. Generalized linear modelling was performed using the 'glm' or 'glm.nb' function from the stats or MASS R packages. Fisher's exact test was performed using the function 'fisher.test' from the stats R package. For each association analysis, we obtained P-values for the main effect using the Wald test and applied multiple hypothesis correction by computing Benjamini-Hochberg false discovery rates (FDRs) and reporting hypotheses with FDR<0.1. Code and source data for generating the main figures and extended data figures is available in the following GitHub repository: `https://github.com/mskilab/loose_ends_2023`

Loose end associations. A negative binomial GLM (glm.nb in MASS R package) was used to model the per-sample burden of specific loose end classes (total, partially mapped, ambiguously mapped), analyzing sample-specific factors, including tumor type, genotype, and presence of a complex event class, as main effects and/or covariates. For tumor type associations, we used a binary indicator of membership in a given tumor type as the main effect, subject to an offset of log total breakends per sample + 1. Tumor types with fewer than 15 cases in the study cohort were excluded, and per sample log breakend count + 1 was used as an offset.

For genotype associations, we used a binary indicator denoting the presence of a mutation (missense mutations, truncating mutation, homozygous deletion) in a given gene as the main effect, with tumor type as a covariate and log total breakend count per sample + 1 as an offset. For this analysis, we considered a list of 451 previously curated DNA damage response genes[26] mutated in at least 25 samples in our study cohort.

For complex SV associations, we used an indicator variable denoting the presence of a specific SV pattern (i.e. chromothripsis, chromoplexy, templated insertion chains (TIC), breakage-fusion-bridge patterns (BFB), tyfonas, double minutes (DM), pyrgo, or rigma) in a sample as the main effect. Tumor type was included as a covariate and log breakend count + 1 per sample was included as an offset.

To associate tumor types with the presence of telomere repeat-positive loose ends, AHR, and NAHR, we used a binomial regression (glm in the stats R package, with argument family = 'binomial'). Here we used nonzero burden of telomere repeat-positive loose ends, putative NAHR loose ends, or putative AHR segments as a binary response variable, and membership in a given tumor type as a binary main effect. Per sample log breakend count + 1 was included as a covariate.

References

1. Hadi, K. *et al.* Distinct classes of complex structural variation uncovered across thousands of cancer genome graphs. *Cell* **183**, 197–210 (2020).

2. Olshen, A. B., Venkatraman, E., Lucito, R. & Wigler, M. Circular binary segmentation for the analysis of array-based dna copy number data. *Biostatistics* **5**, 557–572 (2004).

3. Wala, J. A. *et al.* Svaba: genome-wide detection of structural variants and indels by local assembly. *Genome research* **28**, 581–591 (2018).

4. Deshpande, A., Walradt, T., Hu, Y., Koren, A. & Imielinski, M. Robust foreground detection in somatic copy number data (2019).

5. Ross, E. M., Haase, K., Van Loo, P. & Markowetz, F. Allele-specific multi-sample copy number segmentation in ASCAT. *Bioinformatics* **37**, 1909–1911 (2021).

6. Loo, P. V. *et al.* Allele-specific copy number analysis of tumors. *Proceedings of the National Academy of Sciences* **107**, 16910–16915.

7. Shen, R. & Seshan, V. E. FACETS: allele-specific copy number and clonal heterogeneity analysis tool for high-throughput DNA sequencing. *Nucleic Acids Research* **44**, e131–e131 (2016).

8. Favero, F. *et al.* Sequenza: allele-specific copy number and mutation profiles from tumor sequencing data. *Annals of Oncology* **26**, 64–70 (2015).

9. Ha, G. *et al.* TITAN: inference of copy number architectures in clonal cell populations from tumor whole-genome sequence data. *Genome Research* **24**, 1881 1893 (2014).

10. Kim, S. *et al.* Strelka2: fast and accurate calling of germline and somatic variants. *Nature Methods* **15**, 591–594 (2018).

11. Mallick, S. *et al.* The Simons Genome Diversity Project: 300 genomes from 142 diverse populations. *Nature* **538**, 201 (2016).

12. Cingolani, P. *et al.* A program for annotating and predicting the effects of single nucleotide polymorphisms, SnpEff. *Fly* **6**, 80–92 (2012).

13. Tubio, J. M. *et al.* Extensive transduction of nonrepetitive dna mediated by l1 retrotransposition in cancer genomes. *Science* **345** (2014).

14. Nurk, S. *et al.* The complete sequence of a human genome. *Science* **376**, 44–53 (2022).

15. Jiang, T. *et al.* Long-read-based human genomic structural variation detection with cuteSV. *Genome Biology* **21**, 189 (2020).

16. Elrick, H. *et al.* Abstract LB080: SAVANA: a computational method to characterize structural variation in human cancer genomes using nanopore sequencing. *Cancer Research* **83**, LB080–LB080 (2023).

17. Heller, D. & Vingron, M. SVIM: Structural Variant Identification using Mapped Long Reads. *Bioinformatics* **35**, btz041 (2019).

18. Smolka, M. *et al.* Comprehensive Structural Variant Detection: From Mosaic to Population-Level. *bioRxiv* 2022.04.04.487055 (2022).

19. Ghandi, M. *et al.* Next-generation characterization of the cancer cell line encyclopedia. *Nature* **569**, 503–508 (2019).

20. Spies, N. *et al.* Genome-wide reconstruction of complex structural variants using read clouds. *Nature methods* **14**, 915–920 (2017).

21. Elyanow, R., Wu, H.-T. & Raphael, B. J. Identifying structural variants using linked-read sequencing data. *Bioinformatics* **34**, 353–360 (2018).

22. Fang, L. *et al.* Linkedsv for detection of mosaic structural variants from linked-read exome and genome sequencing data. *Nature communications* **10**, 1–15 (2019).

23. Li, H. Exploring single-sample snp and indel calling with whole-genome de novo assembly. *Bioinformatics* **28**, 1838–1844 (2012).

24. Eberle, M. A. *et al.* A reference data set of 5.4 million phased human variants validated by genetic inheritance from sequencing a three-generation 17-member pedigree. *Genome Research* **27**, 157–164 (2017).

25. Frazer, K. A. *et al.* A second generation human haplotype map of over 3.1 million SNPs. *Nature* **449**, 851–861.

26. Pearl, L. H., Schierz, A. C., Ward, S. E., Al-Lazikani, B. & Pearl, F. M. Therapeutic opportunities within the dna damage response. *Nature Reviews Cancer* **15**, 166–180 (2015).
