# Supplementary material for: Most large structural variants in cancer genomes can be detected without long reads
Source: Nat Genet. 2023 Nov 9;55(12):2139–48. doi: 10.1038/s41588-023-01540-6 (PMC10703688; doi:10.1038/s41588-023-01540-6)
Supplement: Supplementary file 2 — Reporting Summary [file 41588_2023_1540_MOESM2_ESM.pdf]

Corresponding author(s): Marcin Imielinski

Last updated by author(s): Mar 9, 2023

Reporting Summary

Nature Portfolio wishes to improve the reproducibility of the work that we publish. This form provides structure for consistency and transparency in reporting. For further information on Nature Portfolio policies, see our Editorial Policies and the Editorial Policy Checklist.

Statistics

For all statistical analyses, confirm that the following items are present in the figure legend, table legend, main text, or Methods section.

n/a	Confirmed	
☐	☒	The exact sample size (*n*) for each experimental group/condition, given as a discrete number and unit of measurement
☐	☒	A statement on whether measurements were taken from distinct samples or whether the same sample was measured repeatedly
☐	☒	The statistical test(s) used AND whether they are one- or two-sided<br>*Only common tests should be described solely by name; describe more complex techniques in the Methods section.*
☐	☒	A description of all covariates tested
☐	☒	A description of any assumptions or corrections, such as tests of normality and adjustment for multiple comparisons
☐	☒	A full description of the statistical parameters including central tendency (e.g. means) or other basic estimates (e.g. regression coefficient) AND variation (e.g. standard deviation) or associated estimates of uncertainty (e.g. confidence intervals)
☐	☒	For null hypothesis testing, the test statistic (e.g. *F*, *t*, *r*) with confidence intervals, effect sizes, degrees of freedom and *P* value noted<br>*Give P values as exact values whenever suitable.*
☒	☐	For Bayesian analysis, information on the choice of priors and Markov chain Monte Carlo settings
☒	☐	For hierarchical and complex designs, identification of the appropriate level for tests and full reporting of outcomes
☐	☒	Estimates of effect sizes (e.g. Cohen's *d*, Pearson's *r*), indicating how they were calculated

Our web collection on statistics for biologists contains articles on many of the points above.

Software and code

Policy information about availability of computer code

Data collection	BWA MEM (v0.7.17) was used to align short reads, minimap2 (v2.17) was used to align nanopore long reads, and the EMerAld aligner (v0.6.2) was used to align 10X linked reads. Fermi (ttps://github.com/lh3/fermi, v1.1) was used for local assembly of reads near loose ends. SvABA (https://github.com/walaj/svaba/releases/tag/1.1.0, version 1.1.0) was used to call junctions in short read whole genomes. Structural variant calls for linked read whole genome sequencing were identified using NAIBR (https://github.com/raphael-group/NAIBR, commit 15eba96), GROC-SVs (https://github.com/grocsvs/grocsvs, version 0.2.6), and LinkedSV (https://github.com/WGLab/LinkedSV, commit 1b77a14). Structural variant calls for nanopore long read sequencing were identified using cuteSV (https://github.com/tjiangHIT/cuteSV/releases/tag/cuteSV-v2.0.2), SAVANA (https://github.com/cortes-ciriano-lab/savana/releases/tag/0.2.3), SVIM (https://github.com/eldariont/svim/releases/tag/v2.0.0), and Sniffles2 (https://github.com/fritzsedlazeck/Sniffles/releases/tag/v2.0.7).
Data analysis	The data in this study were analyzed using custom open source code that can be accessed from three GitHub repositories: https://github.com/mskilab/loosends , https://github.com/mskilab/JaBbA , and https://github.com/mskilab/gGnome .

For manuscripts utilizing custom algorithms or software that are central to the research but not yet described in published literature, software must be made available to editors and reviewers. We strongly encourage code deposition in a community repository (e.g. GitHub). See the Nature Portfolio guidelines for submitting code & software for further information.

Data

Policy information about availability of data

All manuscripts must include a data availability statement. This statement should provide the following information, where applicable:

- Accession codes, unique identifiers, or web links for publicly available datasets
- A description of any restrictions on data availability
- For clinical datasets or third party data, please ensure that the statement adheres to our policy

SRS and LRS alignments for the LRS cohort have been been deposited at the European Genome-phenome Archive (EGA), which is hosted by the European Bioinformatics Institute (EBI) and the Centre for Genomic Regulation (CRG), under accession number EGAD00001011047. SRS and sLRS alignments for the sLRS breast cancer cohort have been been deposited at EGA under accession number EGAD0000101032. sLRS cell line data were deposited under NCBI Bioproject PRJNA623129. Whole genome profiles for cell lines used in the study were obtained from a previous study (Hadi et al., Cell 2020).

Human research participants

Policy information about studies involving human research participants and Sex and Gender in Research.

Reporting on sex and gender	Sex and gender based analyses were not performed in this study.
Population characteristics	Covariate-based population characteristics of human research populations were not collected for this study.
Recruitment	LRS profiles were generated for twenty two fresh-frozen samples (11 tumor and 11 matched normal tissues) from 11 patients consented for sequencing at Memorial Sloan Kettering Cancer Center (MSKCC, MSKCC IRBs 00-144, 12-245, and 16-675). Cases comprised 10 melanomas and one triple negative breast cancer. Synthetic long read sequencing (sLRS) whole genome profiling was performed on 25 breast ductal carcinoma cases (25 tumor and 25 matched normal tissue) from MSKCC consented for sequencing under MSKCC IRB (16-675).
Ethics oversight	Memorial Sloan Kettering Cancer Center Institutional Review Board

Note that full information on the approval of the study protocol must also be provided in the manuscript.

Field-specific reporting

Please select the one below that is the best fit for your research. If you are not sure, read the appropriate sections before making your selection.

☒ Life sciences ☐ Behavioural & social sciences ☐ Ecological, evolutionary & environmental sciences

For a reference copy of the document with all sections, see nature.com/documents/nr-reporting-summary-flat.pdf

Life sciences study design

All studies must disclose on these points even when the disclosure is negative.

Sample size	No sample size calculation was performed. Sample size was determined by availability of tumor and matched normal with adequate quality and quantity for short read, long read, and linked read whole genome profiling, and availability of previously published whole genome profiles.
Data exclusions	Samples with low (<=0.5) purity calculated after whole genome sequencing were excluded from the analysis.
Replication	Replicates are noted in the associated figure panel captions. Pan-cancer SRS results were replicated using additional SRS and LRS / sLRS profiling. Code and source data for analyses and figure panels have been made available: https://github.com/mskilab/loose_ends_2023.
Randomization	Randomization was not applicable to this study as the goal was not to evaluate the causal effect of an intervention and samples were not divided into groups with/without an intervention.
Blinding	Blinding was not applicable to this study as the goal was not to evaluate the causal effect of an intervention and samples were not divided into groups with/without an intervention.

Reporting for specific materials, systems and methods

We require information from authors about some types of materials, experimental systems and methods used in many studies. Here, indicate whether each material, system or method listed is relevant to your study. If you are not sure if a list item applies to your research, read the appropriate section before selecting a response.

Materials & experimental systems

n/a	Involved in the study
☒	☐ Antibodies
☐	☒ Eukaryotic cell lines
☒	☐ Palaeontology and archaeology
☒	☐ Animals and other organisms
☒	☐ Clinical data
☒	☐ Dual use research of concern

Methods

n/a	Involved in the study
☒	☐ ChIP-seq
☒	☐ Flow cytometry
☒	☐ MRI-based neuroimaging

Eukaryotic cell lines

Policy information about cell lines and Sex and Gender in Research

Cell line source(s)	Cell lines HCC1943 (CRL-2338), HCC1954BL (CRL-2339) HCC1143 (CRL-2321), HCC1143BL (CRL-2362), U2OS (HTB-96), NCI-H838 (CRL-5844), NCI-H661 (HTB-183), SAOS2 (HTB-85) are available through ATCC, Manassas, VA.
Authentication	All cell lines were purchased from validated sources and whole genome sequenced.
Mycoplasma contamination	All cell lines tested negative for mycoplasma contamination.
Commonly misidentified lines (See ICLAC register)	No commonly misidentified cell lines were used.
